# The innovation of the symbiosome has enhanced the evolutionary stability of nitrogen fixation in legumes

**DOI:** 10.1111/nph.18321

**Published:** 2022-07-28

**Authors:** Sergio M. de Faria, Jens J. Ringelberg, Eduardo Gross, Erik J. M. Koenen, Domingos Cardoso, George K. D. Ametsitsi, John Akomatey, Marta Maluk, Nisha Tak, Hukam S. Gehlot, Kathryn M. Wright, Neung Teaumroong, Pongpan Songwattana, Haroldo C. de Lima, Yves Prin, Charles E. Zartman, Janet I. Sprent, Julie Ardley, Colin E. Hughes, Euan K. James

**Affiliations:** ^1^ Embrapa Agrobiologia 465 km 07, Seropédica Rio de Janeiro BR 23891‐000 Brazil; ^2^ Department of Systematic and Evolutionary Botany University of Zurich Zollikerstrasse 107 Zurich CH‐8008 Switzerland; ^3^ Departamento de Ciências Agrárias e Ambientais Universidade Estadual de Santa Cruz (UESC) Ilhéus BA 45662‐900 Brazil; ^4^ National Institute of Science and Technology in Interdisciplinary and Transdisciplinary Studies in Ecology and Evolution (INCT IN‐TREE) Instituto de Biologia, Universidade Federal de Bahia (UFBA) Rua Barão de Jeremoabo, s.n., Ondina Salvador 40170‐115 BA Brazil; ^5^ CSIR‐Forestry Research Institute of Ghana FUMESUA PO Box UP 63 KNUST Kumasi Ghana; ^6^ The James Hutton Institute Invergowrie Dundee DD2 5DA UK; ^7^ Department of Botany, BNF and Microbial Genomics Lab. Center of Advanced Study, Jai Narain Vyas University Jodhpur 342001 Rajasthan India; ^8^ School of Biotechnology, Institute of Agricultural Technology Suranaree University of Technology Nakhonratchasima 30000 Thailand; ^9^ Instituto de Pesquisas Jardim Botânico do Rio de Janeiro (JBRJ/MMA) Rua Pacheco Leão 915 Rio de Janeiro 22460‐030 RJ Brazil; ^10^ Instituto Nacional da Mata Atlântica (INMA‐MCTI) Av. José Ruschi 4 Santa Teresa 29650‐000 ES Brazil; ^11^ CIRAD, UMR LSTM Campus de Baillarguet 34398 Montpellier Cedex 5 France; ^12^ Departamento de Biodiversidade Instituto Nacional de Pesquisas da Amazônia (INPA) Av. André Araújo Aleixo, Caixa Postal 478 Manaus 69060‐001 AM Brazil; ^13^ Division of Plant Sciences University of Dundee at The James Hutton Institute Invergowrie Dundee DD2 5DA UK; ^14^ College of Science, Health, Engineering and Education Murdoch University Murdoch WA 6150 Australia

**Keywords:** evolution, fixation threads, Leguminosae, nitrogen fixation, nodulation, phylogenomics, symbiosis, symbiosomes

## Abstract

- Nitrogen‐fixing symbiosis is globally important in ecosystem functioning and agriculture, yet the evolutionary history of nodulation remains the focus of considerable debate. Recent evidence suggesting a single origin of nodulation followed by massive parallel evolutionary losses raises questions about why a few lineages in the N_2_‐fixing clade retained nodulation and diversified as stable nodulators, while most did not. Within legumes, nodulation is restricted to the two most diverse subfamilies, Papilionoideae and Caesalpinioideae, which show stable retention of nodulation across their core clades.
- We characterize two nodule anatomy types across 128 species in 56 of the 152 genera of the legume subfamily Caesalpinioideae: fixation thread nodules (FTs), where nitrogen‐fixing bacteroids are retained within the apoplast in modified infection threads, and symbiosomes, where rhizobia are symplastically internalized in the host cell cytoplasm within membrane‐bound symbiosomes (SYMs).
- Using a robust phylogenomic tree based on 997 genes from 147 Caesalpinioideae genera, we show that losses of nodulation are more prevalent in lineages with FTs than those with SYMs.
- We propose that evolution of the symbiosome allows for a more intimate and enduring symbiosis through tighter compartmentalization of their rhizobial microsymbionts, resulting in greater evolutionary stability of nodulation across this species‐rich pantropical legume clade.

## Introduction

The N_2_‐fixing clade of angiosperms includes all plants that form specialized organs known as nodules, within which they house intracellular diazotrophic bacteria (van Velzen *et al*., 2018a). Within this clade, some species of Cucurbitales, Fagales and Rosales engage in nodulating symbiosis with the filamentous actinobacteria *Frankia*, while *Parasponia* (Rosales, Cannabaceae) and legumes (Fabales, Fabaceae) host phylogenetically diverse strains of α‐ and β‐proteobacteria collectively known as rhizobia (Soltis *et al*., 1995; Sprent *et al*., 2017; Griesmann *et al*., 2018; van Velzen *et al*., 2018a). Strikingly, nodulation is mostly a rare trait across these four orders, having been reported in relatively few species except in Fabales, where the majority of the *c*. 20 000 species in the Fabaceae appear to be nodulated (Doyle, 2011). Across the legume family, nodulation is also very unevenly distributed, with most species in Papilionoideae and the Mimosoid clade (Caesalpinioideae *sensu* LPWG, 2017) being nodulated, whereas nodulation is less common in nonmimosoid Caesalpinioideae and absent in the other four smaller legume subfamilies (*sensu* LPWG, 2017). The reasons for this uneven phylogenetic distribution of nodulation are unclear.

Despite the ecological and economic significance of N_2_‐fixing root nodule symbiosis in ecosystem functioning and agriculture (Peoples *et al*., 1995; Batterman *et al*., 2013; Vitousek *et al*., 2013; Epihov *et al*., 2017), there is no consensus about the evolutionary origins of this important trait. Hypotheses have shifted from a scenario of multiple origins (Doyle, 2011; Werner *et al*., 2014), potentially predisposed by a cryptic precursor that evolved in the ancestor of the N_2_‐fixing clade (Soltis *et al*., 1995; Werner *et al*., 2014), to one of a single origin and massive parallel evolutionary losses (Soltis *et al*., 1995; Griesmann *et al*., 2018; van Velzen *et al*., 2018a,b). This second hypothesis was generally dismissed because multiple independent origins provided a more parsimonious solution for the phylogenetic distribution of nodulating lineages, and because variation in nodule types and microsymbionts suggested that nodules are potentially nonhomologous and arose multiple times. This clustered homoplasious occurrence of nodulation, confined to just one clade of angiosperms (Marazzi *et al*., 2012), prompted the idea that a cryptic precursor evolved in the ancestor of the N_2_‐fixing clade, which conferred a propensity for nodulation that was expressed in just a subset of lineages (Soltis *et al*., 1995; Doyle, 2011, 2016; Werner *et al*., 2014). However, no evidence for such a precursor, genetic or otherwise, has been found (Doyle, 2016; Griesmann *et al*., 2018; van Velzen *et al*., 2018a). Furthermore, nodulation involves structural and biochemical innovations underpinned by many genes, multiple developmental and signalling pathways, and coordination between the host and the microsymbiont (Brewin, 2004; Oldroyd & Downie, 2008; Oldroyd, 2013; Sprent *et al*., 2017; Ardley & Sprent, 2021; Ledermann *et al*., 2021), such that evolutionary gains of nodulation are likely to be more difficult than losses (van Velzen *et al*., 2018a; Edwards, 2019). Recently, the alternative hypothesis of a single evolutionary origin of nodulation followed by numerous parallel evolutionary losses has gained traction, notably from comparative genomic studies documenting pseudogenization or loss of key nodulation genes in nonnodulating species, indicative of secondary losses of nodulation (Griesmann *et al*., 2018; van Velzen *et al*., 2018a; Zhao *et al*., 2021). Reexamination of the structural and developmental homologies and commonalities in symbiotic gene function across nodulating lineages spanning the N_2_‐fixing clade suggested that these also provide more compelling evidence for the single gain and multiple losses hypothesis (van Velzen *et al*., 2018a; Shen & Bisseling, 2020).

This shift in thinking prompts questions about how the numerous secondary losses of nodulation are distributed across lineages and through time, why certain lineages retained nodulation to diversify as stable N_2_‐fixers whereas many others lost this trait, and why, through time, N_2_‐fixing symbiosis apparently became nonadvantageous for the large majority of N_2_‐fixing clade lineages.

One trait that has not been considered as a potential determinant of evolutionary stability of nodulation is the occurrence of two distinct anatomical arrangements of N_2_‐fixing bacteria within the nodule. In the majority of papilionoid legumes, such as pea and *Medicago*, an infection thread (IT), formed from invagination of a root hair cell wall, conveys rhizobia from the point of infection to the nodule primordium. Rhizobia within the IT are budded off once they reach the nodule cell and are retained within it only by the host plasmalemma‐derived symbiosome (or peribacteroid) membrane, where they differentiate into their N_2_‐fixing bacteroid forms (Sprent, 2001, 2009; Brewin, 2004; Sprent *et al*., 2017; Parniske, 2018; Ardley & Sprent, 2021; Tsyganova *et al*., 2021). By contrast, in all actinorhizal symbioses and in a subset of nodulating legumes the N_2_‐fixing bacteria are retained within modified, thin‐walled, infection threads called fixation threads (FTs), remaining enclosed within the plant cell wall and the plasmalemma. Hereafter, we refer to these as FT‐type nodules, and those in which the bacteroids are enclosed in symbiosomes as SYM‐type nodules.

FT‐type nodules were first described in actinorhizal plants (enclosing their *Frankia* microsymbionts), and in *Parasponia*, the only nonlegume known to form nodules with rhizobia (Trinick, 1980; Lancelle & Torrey, 1985; Smith *et al*., 1986). They were later observed in legumes, mostly in woody Caesalpinioideae (*sensu* LPWG, 2017) where they appeared to be relatively common (de Faria *et al*., 1986, 1987; Naisbitt *et al*., 1992; Sprent, 2001; Fonseca *et al*., 2012). FT‐type nodules have also been reported in a few legumes belonging to the subfamily Papilionoideae, which is sister to Caesalpinioideae (Koenen *et al*., 2020b; Zhao *et al*., 2021), including tree genera such as *Andira*, *Dahlstedtia* and *Hymenolobium*, and members of tribe Brongniartieae (de Faria *et al*., 1986, 1987; Sprent, 2001, 2009; Sprent *et al*., 2013, 2017). Ultrastructural and histochemical analyses of FT‐type nodules in *Parasponia* with rhizobia (Smith *et al*., 1986), actinorhizal nodules with *Frankia* (Pawlowski & Demchenko, 2012) and in some legumes (de Faria *et al*., 1986, 1987; Naisbitt *et al*., 1992) revealed that FTs are superficially similar to the cell wall‐bound ‘invasive’ IT, for example in harbouring some pectin (Fonseca *et al*., 2012). The IT is an extension of the host cell wall and comprises mainly cellulose and pectin; its role appears to be largely protective, preventing the bacteria from invading the plant in a disorganized or pathogenic manner (Brewin, 2004; Tsyganova *et al*., 2021). However, the composition and role of the FT remain uncertain. Moreover, the precise nature of the FT in relation to the symbiosome membrane, which in SYM‐type nodules is essential for the exchange of nutrients between the host cytoplasm and the bacteroid (White *et al*., 2007), remains unknown.

**Fig. 1:**
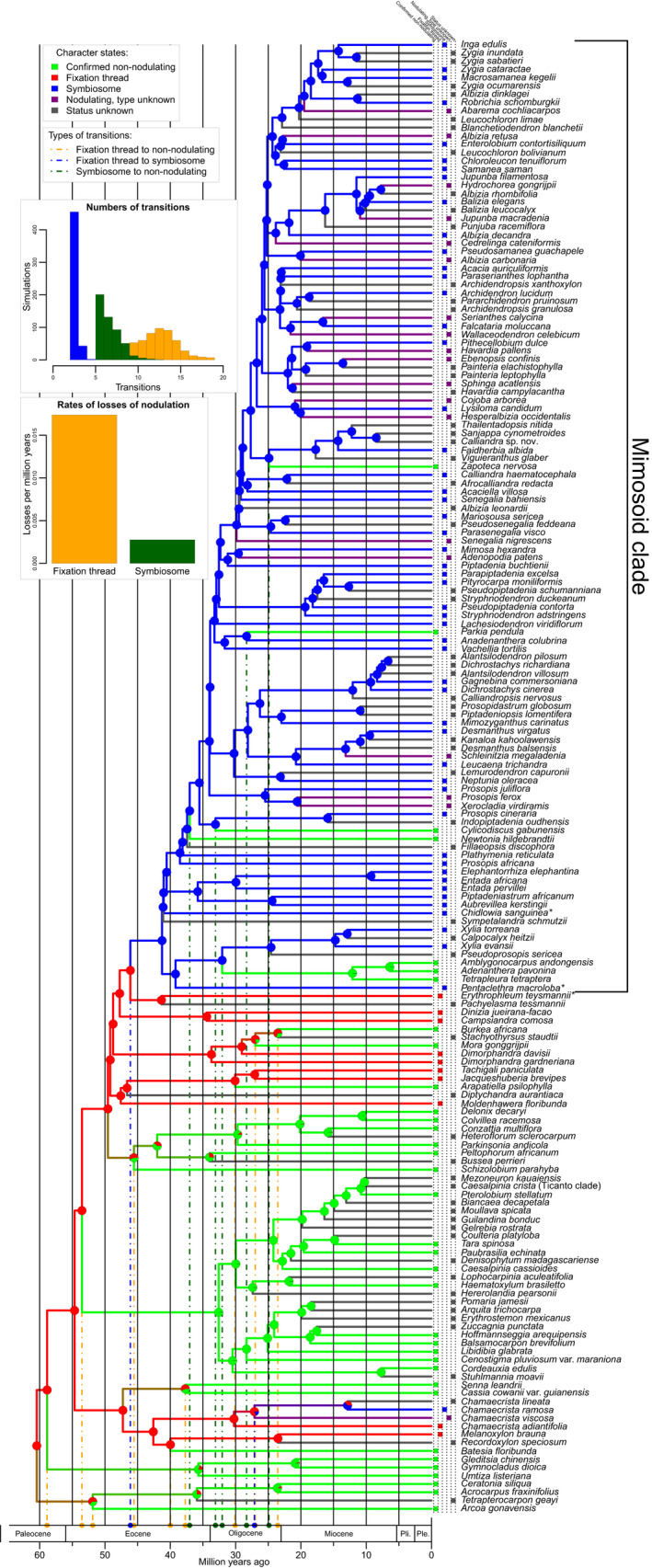
Evolutionary trajectories of nodulation and nodule type across a time‐calibrated phylogeny of the legume subfamily Caesalpinioideae. Pie charts on nodes show the proportions of the most probable reconstructed character states: nonnodulating, fixation thread (FT‐type nodules), symbiosome (SYM‐type nodules), nodulating but of unknown type, and nodulation status unknown, summarized over 500 simulations. Branch colours denote the nodulation status of the node or tip it subtends and the coloured boxes in front of each taxon name show the character state for that species. Note that in three clades (the *Senna* + *Cassia* clade, the *Arcoa* + *Acrocarpus* clade and the *Peltophorum* clade) ‘double’ losses of FT‐type nodules are inferred to have occurred simultaneously in both descendant lineages of that node. For example, the crown node of the *Senna* + *Cassia* clade is inferred to be nodulating with FT‐type nodules even though *Senna* and *Cassia* are nonnodulating. The dashed orange, blue and dark green vertical lines show the phylogenetic locations and maximum ages of the various character state transitions on the tree. Using the same colours, the histograms show the frequencies of the number of transitions from FT to SYM (blue), from SYM to nonnodulation (green) and from FT to nonnodulation (orange), and the rates of losses of nodulation per million years for SYM to nonnodulation (green) and FT to nonnodulation (orange) across 500 independent character estimations. Note that while the three other character state transitions, from nonnodulating to FT or SYM‐type nodules, and from SYM to FT, were not allowed under our model, and were therefore fixed at zero, an alternative model permitting all character state transitions gave an identical result (Supporting Information Fig. S3). Asterisks after terminal names indicate species which are the focus of detailed nodule anatomical work presented in Figs 2, 3, 4. Ple, Pleistocene; Pli, Pliocene.

Within legumes, nodulation is restricted to the two largest subfamilies, Caesalpinioideae (which includes the phylogenetically nested Mimosoid clade, formerly subfamily Mimosoideae – see LPWG, 2017) and Papilionoideae (Sprent *et al*., 2017; Ardley & Sprent, 2021). Here we investigate the occurrence of FT‐ and SYM‐type nodulation across Caesalpinioideae, the second largest subfamily of legumes, with 152 genera and *c*. 4600 species distributed pantropically across all lowland tropical biomes, with minor incursions into temperate regions. We provide an updated census of nodulation occurrence, including three new records in genera of previously unknown nodulation status, plus extensive new data regarding FT‐ and SYM‐type nodules across genera. We investigate whether these two nodule types represent different degrees of ‘compartmentalization’ (i.e. physical structures that allow hosts to spatially separate symbionts; Chomicki *et al*., 2020), by examining the anatomy and structure of nodules in *Chidlowia*, *Pentaclethra* and *Erythrophleum*, genera that span an evolutionary transition from FT‐ to SYM‐type nodules that we hypothesize to have occurred within Caesalpinioideae along the branch subtending the Mimosoid clade (Fig. 1). *Erythrophleum*, which is placed in the sister clade of the Mimosoid clade, has FT‐type nodules, while *Pentaclethra* and *Chidlowia*, which are among the first‐branching lineages of the Mimosoid clade (Manzanilla & Bruneau, 2012; Koenen *et al*., 2020a), as well as all other studied taxa in the Mimosoid clade, have SYM‐type nodules.

We test the hypothesis that the transition in nodule anatomy from FT‐ to SYM‐type nodules constituted an evolutionary innovation that led to more stable retention of nodulation, whereas lineages in which FT‐type nodules occur are more prone to evolutionary losses of nodulation. For this, we explore the number and phylogenetic distribution of evolutionary losses of nodulation using a robust phylogenomic backbone that includes 97% of Caesalpinioideae genera. We also examined the composition of the FT wall in more detail than has hitherto been achieved using immunohistochemical methods that have been used for ITs (Brewin, 2004; Tsyganova *et al*., 2021) in order to better elucidate its possible role in symbiosis.

## Materials and Methods

### Nodulation and nodule anatomy

Basic nodulation data (nodulated or nonnodulated) were obtained from Sprent (2001, 2009), from papers or reports published since 2009, and from previously unpublished records in the databases of the authors, including new reports of nodulation status (Supporting Information Tables S1 and S2). Where no data are available the nodulation status of a genus is listed as Uncertain (Un) (Table S1).

Anatomical types (FT or SYM) were determined from extensive data newly obtained here (Tables S1 and S2) alongside published data for the specific taxa that were used to construct the phylogeny, or related species in the same genus, based on substantial data that indicate that nodulation is almost always a generic trait. However, losses of nodulation have been reported within the genus *Senegalia* (see below), and it should be acknowledged that the relatively limited nodulation record and sampling in the Caesalpinioideae may hide additional intrageneric losses (Sprent *et al*., 2017). All samples were prepared for light and electron microscopy according to de Faria *et al*. (1986, 1987) and Fonseca *et al*. (2012), unless otherwise stated in Table S2; in total, samples from 95 species were newly prepared for this study. Additional samples of nodules from *Chidlowia sanguinea*, *Entada polystachya*, *Erythrophleum* spp., *Moldenhawera* spp. and *Pentaclethra macroloba* were prepared specifically to examine in detail the presence of FTs or symbiosomes (Table S2, Notes S1). For these samples, slices from four or more nodules per species were fixed in 2.5% glutaraldehyde and processed in two ways: (1) for light microscopy and immunogold transmission electron microscopy (TEM) with the monoclonal antibodies JIM5 and JIM7, which recognize, respectively, unesterified and esterified pectin (VandenBosch *et al*., 1989; Tsyganova *et al*., 2021), according to Fonseca *et al*. (2012); and (2) for identifying the symbiosome membrane by TEM using additional postfixation in osmium tetroxide followed by embedding in epoxy resin according to Rubio *et al*. (2009). Ultramicrotomy, staining of sections for light microscopy and for TEM, and immunogold labelling with JIM5 for TEM were as described in Fonseca *et al*. (2012).

For immunohistochemical analysis of the FT wall, confocal laser scanning microscopy (CLSM) was performed on slides containing semithin sections (1 μm thickness) of *Erythrophleum* and *Pentaclethra* nodules fixed and embedded as per method (1) above. The sections were incubated for 2 h in 1 : 10 dilutions of monoclonal antibodies raised against various plant cell wall components (all obtained from Plant Probes, Centre for Plant Sciences, University of Leeds, UK): Lm2, which labels β‐linked‐GlcA in arabinogalactose protein (AGP) glycan; Lm5, which labels the pectic polysaccharide rhamnogalacturonan; and Lm15, which labels the XXXG motif of the nonpectic, noncellullosic polysaccharide xyloglucan. Then, after washing twice in distilled water (dH_2_O) the sections were incubated for 1 h in a 1 : 500 dilution of goat antirat Alexa 488 secondary antibody (ThermoFisher, Loughborough, UK) followed by several rinses with dH_2_O. After mounting in coverslips and Fluoromount (ThermoFisher), the sections were examined using a Zeiss LSM 710 confocal laser scanning microscope (Carl Zeiss Microscopy Ltd, Cambourne, UK), fitted with a W Plan‐Apochromat 40× lens, using spectral imaging with excitation at 488 nm and emissions between 494 nm and 727 nm. The images were colour‐coded according to wavelength and enhanced using the Min/Max function in Zen 2010 software. Ultrathin sections (80 nm) of the same samples were then immunogold labelled for TEM using the same monoclonal antibodies as those for CLSM (Lm2, Lm5, Lm15) according to Fonseca *et al*. (2012).

### Phylogeny and ancestral trait estimation

We used a recently constructed time‐calibrated phylogeny of Caesalpinioideae that included 147 of the 152 genera, which was based on DNA sequence data derived from targeted enrichment of 997 nuclear genes selected specifically for phylogenomics of the Mimosoid clade using the Mimobaits bait set (Koenen *et al*., 2020a) to generate a large phylogenomic Hybseq dataset (Ringelberg *et al*., 2022). Using this much larger gene set allowed us to overcome lack of resolution prevalent across the backbone of the nonmimosoid grade in previous phylogenies that were based on much smaller traditional Sanger DNA sequence datasets (Bruneau *et al*., 2008; Manzanilla & Bruneau, 2012; LPWG, 2017) to generate a robust and densely sampled phylogenetic hypothesis. The five unsampled genera are *Hultholia*, a member of the Caesalpinia clade (Gagnon *et al*., 2016), a group of 27 genera that are all either nonnodulating or of unknown nodulation status; *Stenodrepanum*, which is sister to *Hoffmannseggia*, and is also placed in the Caesalpinia clade (Gagnon *et al*., 2016); *Pterogyne*, which is also nonnodulating and probably forms a phylogenetically isolated monogeneric lineage in the nonmimosoid grade of Caesalpinioideae that is potentially sister to a large clade comprising all Caesalpinioideae except the Umtiza and Ceratonia clades (Zhao *et al*., 2021); *Microlobius*, which is nodulating with SYM‐type nodules and probably nested within the genus *Stryphnodendron* (Simon *et al*., 2016; Ribeiro *et al*., 2018); and finally the nonmimosoid *Vouacapoua*, which is nonnodulating, but is probably placed in the Cassia clade, which contains both nodulating and nonnodulating lineages (Bruneau *et al*., 2008).

The original 420‐taxon Caesalpinioideae phylogeny of Ringelberg *et al*. (2022) was time‐calibrated in beast (Drummond & Rambaut, 2007), using a species tree topology estimated by astral (Zhang *et al*., 2018), a subset of 100 informative and clock‐like genes, and seven Caesalpinioideae fossil constraints (Ringelberg *et al*., 2022). We pruned this chronogram until each genus was represented by just a single taxon, with two exceptions: first, the genus *Chamaecrista*, for which we retained four species due to known variation in nodule type within that genus (Naisbitt *et al*., 1992; Santos *et al*., 2017); and second, the dense sampling of 420 taxa in the original phylogeny allowed us to test the monophyly of genera, and for nonmonophyletic genera we retained representative taxa for each para‐/polyphyletic lineage. Nodulation data (Table S1) were matched to the tips of this tree in as conservative a way as possible. For example, in the case of *Prosopis*, which is polyphyletic and is thus represented by four taxa in the tree, these were scored as follows: *P. juliflora*, *P. cineraria* and *P. africana*, which represent three independent lineages, are scored as SYM, as the nodule type of these taxa is known. By contrast, the taxon representing the fourth lineage, *P. ferox*, is scored as nodulating but with an unknown nodule type, because *P. ferox* and other taxa in this clade are nodulating, but their nodule types are unknown. In the case of *Senegalia*, which is also nonmonophyletic and where losses of nodulation have been reported (Sprent, 2001, 2009), but where the phylogenetic distribution of those losses remains unknown, we ran an additional trait reconstruction including two losses within the genus. The extensive generic nonmonophyly visible in Caesalpinioideae, especially within the mimosoid clade (Fig. 1), is the focus of current taxonomic work reported elsewhere (J. J. Ringelberg *et al*., unpublished).

Nodulation status was estimated across this phylogeny using a model with three character states: nonnodulating, fixation thread (FT), and symbiosome (SYM). We explicitly followed the single gain, multiple losses evolutionary model for the origins of nodulation; that is, nodulation evolved only once in angiosperms and was subsequently lost many times. To do this, we constrained the model so that the root state of the tree was set to FT and ran the reconstructions either allowing only three types of transitions: from FT to nonnodulating, from FT to SYM, and from SYM to nonnodulating, or allowing all possible transitions. We used stochastic character mapping implemented in the function make.simmap in the phytools R package (Revell, 2012) to simulate 500 independent evolutionary trajectories of nodulation across the time‐calibrated phylogeny. Results were summarized onto this tree across all simulations, with transitions inferred along branches connecting nodes that have different character states in the majority of the 500 simulations. Rates of character change (i.e., events per million years) were extracted from the transition matrix of the continuous‐time‐reversible Markov model fitted to the data by the make.simmap function. Taxa for which the nodulation status is unknown were assigned equal probabilities for all three character states.

## Results

### Nodulation and nodule anatomy

Data on nodule anatomy for 128 species of 56 genera of Caesalpinioideae, including more than 80 records newly reported here (Tables S1, S2), show that no species belonging to the Mimosoid clade have FT‐type nodules; that is, all known nodulating mimosoids have SYM‐type nodules (Figs 1, S1). By contrast, all nodulating species from the grade subtending the Mimosoid clade have FT‐type nodules (Figs 1, S2), except for a subset of species of *Chamaecrista*. In that sense *Chamaecrista* is exceptional among the taxa of the nonmimosoid grade, as it harbours species with either FT‐ or SYM‐type nodules (Naisbitt *et al*., 1992).

**Fig. 2:**
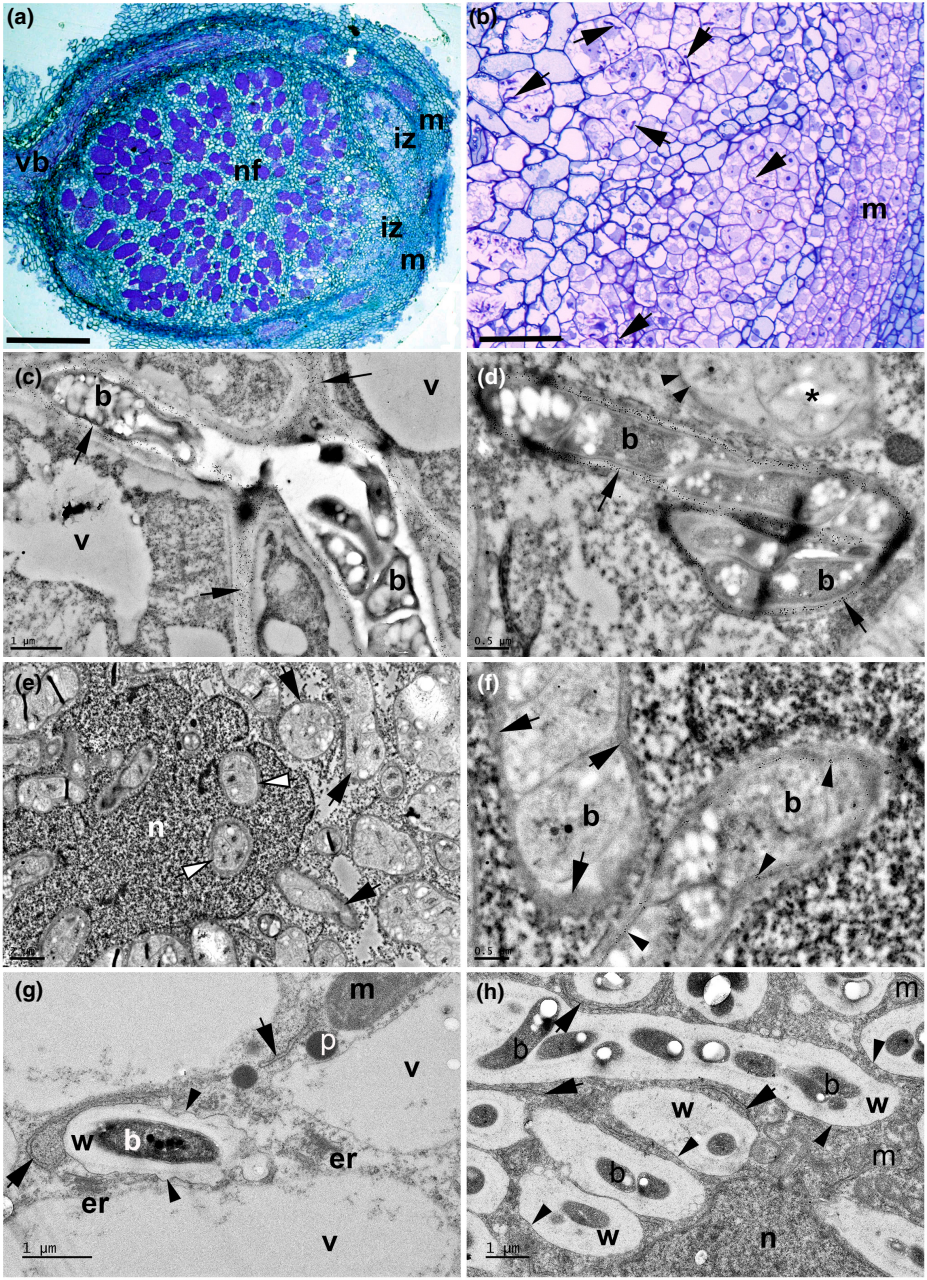
Nonmimosoid grade caesalpinioid nodules in the genus *Erythrophleum* contain bacteroids enclosed within fixation threads (FTs). Light (a, b) and transmission electron microscope (c–h) images of sections of nodules from *E. ivorense* (a–f) and *E. suaveolens* (g, h). (a) Whole nodule longitudinal profile illustrating the zonation typical of an indeterminate nodule (m, meristem; iz, invasion zone; nf, nitrogen fixing zone). Bar, 250 μm. (b) Higher magnification view of the iz in which newly divided host cells derived from the meristem (m) are being invaded by numerous infection threads (arrows). Bar, 10 μm. (c) Large infection thread containing bacteria (b) invading cells in the iz; the walls of the infection thread are densely immunogold labelled with 10 nm gold particles linked to JIM5 (arrows), a monoclonal antibody which recognizes nonesterified pectin. v, vacuole. Bar, 1 μm. (d) Infection thread in the iz–nf boundary with its cell walls labelled with JIM5 (large arrows) adjacent to an FT (*) with a thinner cell wall that is very sparsely labelled with JIM5 (arrowheads). Bar, 500 nm. (e) Cell in the nf zone packed with FTs (black arrows), including within the nucleus (n) indicated by white arrowheads. Bar, 2 μm. (f) Detail of FTs in the nf zone containing N‐fixing bacteroids (b); the FT walls range from being sparsely labelled with JIM5 (arrowheads) to exhibiting little or no obvious labelling (single gold particles are indicated by arrows). Bar, 500 nm. (g) High‐resolution image of a bacteroid (b) forming within a strand of cytoplasm between vacuoles (v) in an iz cell; the bacteroid is surrounded by a cell wall (w) that is being enveloped in a membrane (arrowheads), stretches of which (arrows) appear to be derived from nearby endoplasmic reticulum/Golgi bodies (er). The intense metabolic activity of this process is suggested by the nearby mitochondria (m) and peroxisomes (p). Bar, 1 μm. (h) Bacteroids (b) in newly formed FTs packed into a new N‐fixing cell in the early nf zone adjacent to the iz; the bacteroids are surrounded by the FT wall (w), which is itself surmounted by a symbiosome membrane (arrowheads). Note the membranes within the cytoplasm that are associated with the FTs (arrows). n, nucleus; m, mitochondrion. Bar, 1 μm.

Mature nodules of *Erythrophleum ivorense* and *E. suaveolens*, placed in the sister group of the Mimosoid clade (Fig. 1), are of the indeterminate type (Sprent, 2001, 2009; Sprent *et al*., 2017), and similar to those described in detail in the genus *Dimorphandra*, which is related to *Erythrophleum* (Fonseca *et al*., 2012). Accordingly, as with *Dimorphandra*, nodules have a meristem at the tip and a large invasion zone (IZ) (Fig. 2a), containing cells being invaded by rhizobia (Fig. 2b), and an N_2_‐fixing zone occupying most of the nodule volume; this contains a mix of infected and uninfected cells (Fig. 2a). Bacteria can be seen to invade the IZ cells via ITs emerging from between the cells (Fig. 2c); these ITs are intensely immunogold‐labelled with the monoclonal antibody JIM5, indicating that they contain unesterified pectin, specifically partially methylated homogalacturonan (VandenBosch *et al*., 1989; Tsyganova *et al*., 2021), and are similar in that respect to the host cell wall (Fig. 2c,d). Similar results were obtained with JIM7 (data not shown), indicating that *Erythrophleum* ITs also contain esterified pectin in common with papilionoid nodules (Tsyganova *et al*., 2021). Infected cells in the N_2_‐fixing zone contain numerous FTs (Fig. 2e), bound by cell walls that are thinner than those of the ITs, and either have sparser labelling with JIM5 (Fig. 2d,f) and JIM7 (not shown) or have no apparent labelling (Fig. 2f). Higher definition TEM with osmicated samples reveals that the FTs are surrounded by a cell membrane (Fig. 2g,h), which appears to be derived from the host endoplasmic reticulum (ER). This shows that the FT comprises a multilayered compartment consisting of a cell membrane, the FT cell wall, the lumen of the FT and the bacteroid (Fig. 2g). Infected cells of nodules of the neotropical legumes *Moldenhawera floribunda* and *M. blanchetiana* var. *multijuga*, also placed in the nonmimosoid grade of caesalpinioids (Fig. 1), are packed with bacteroids enclosed within FTs (Fig. S2a,b). As with *Erythrophleum*, the ITs are intensely labelled with JIM5 (Fig. S2c), but the FTs considerably less so (Fig. S2d); the FTs in *Moldenhawera* are also associated with membranes arising from the ER (Fig. S2e,f). Another three nonmimosoid neotropical caesalpinioid genera (*Jacqueshuberia purpurea*, Fig. S2g,h; *Tachigali rugosa*, Fig. S2i,j; and *Campsiandra comosa*, Fig. S2k,l) also have their bacteroids enclosed in FTs labelled to various degrees with JIM5. Together with published reports on *Chamaecrista* (de Faria *et al*., 1987; Naisbitt *et al*., 1992) and *Dimorphandra* (Fonseca *et al*., 2012), these observations of five additional genera demonstrate the ubiquity of FT‐type nodules across nodulating lineages in the nonmimosoid grade of Caesalpinioideae (Fig. 1).

**Fig. 3:**
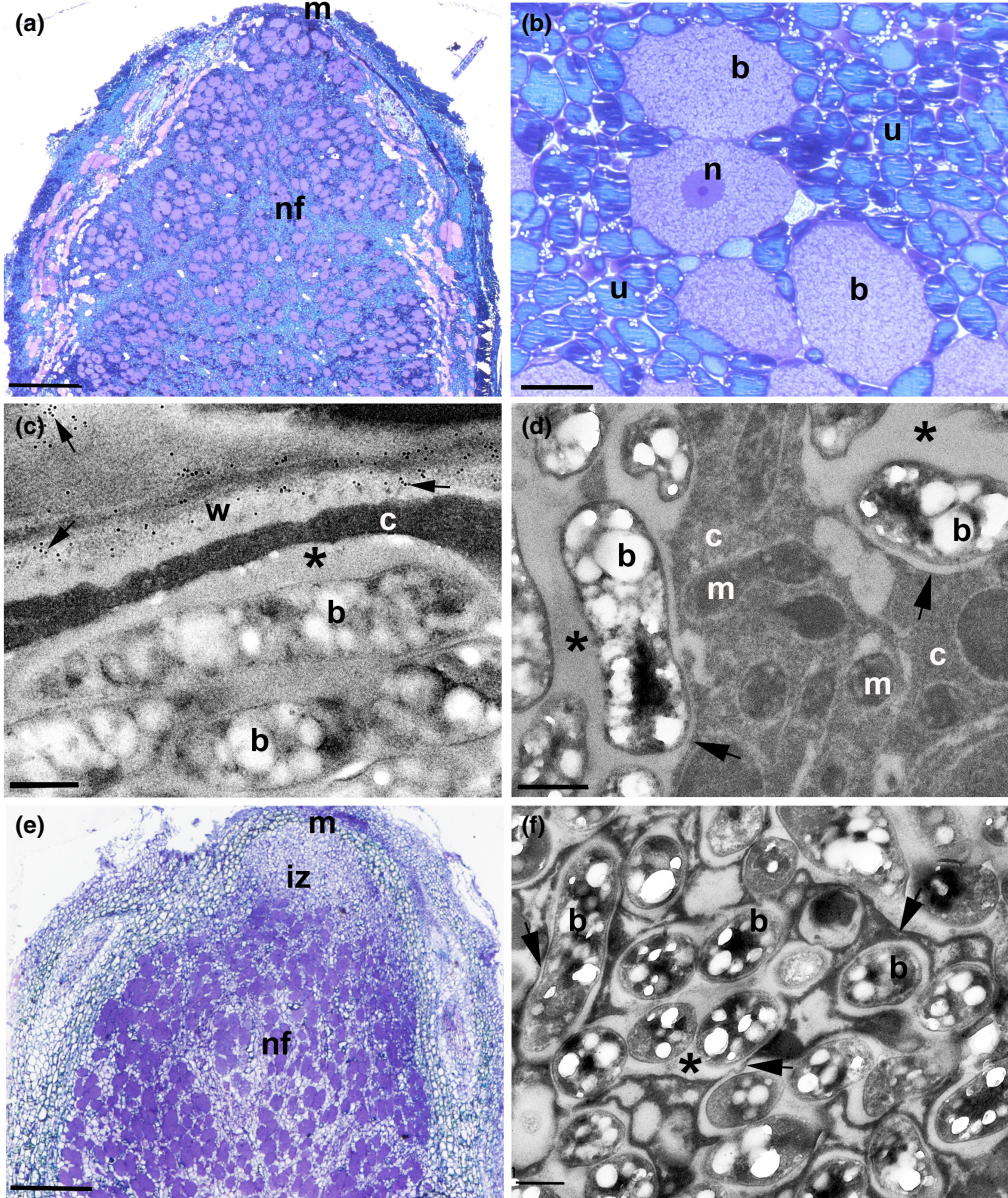
Nodules of caesalpinioids from the Mimosoid clade contain bacteroids enclosed within symbiosomes (SYMs). Light (a, b, e) and transmission electron microscope (c, d, f) images of sections of nodules from *Pentaclethra macroloba* (a–d) and *Chidlowia sanguinea* (e, f). (a) Whole *P. macroloba* nodule longitudinal profile illustrating the zonation typical of an indeterminate nodule (m, meristem; nf, nitrogen fixing zone). Bar, 500 μm. (b) Higher magnification view of the nf zone showing large bacteroid‐containing cells (b) surrounded by smaller and more numerous uninfected cells (u). n, nucleus. Bar, 25 μm. (c) Bacteroids (b) within a symbiosome adjacent to the host cell wall (w) which is immunogold labelled with 10 nm gold particles linked to JIM5 (arrows). The symbiosome peribacteroid space is marked with an asterisk (*); note that there is no cell wall separating the symbiosome from the host cytoplasm (c). Bar, 500 nm. (d) High‐resolution image of bacteroids (b) housed in symbiosomes; the symbiosome membrane separating it from the cytoplasm (c) are marked with arrows, and the peribacteroid space by an asterisk (*). m, mitochondrion. Bar, 500 nm. (e) Whole *C. sanguinea* nodule longitudinal profile illustrating the zonation typical of an indeterminate nodule (m, meristem; iz, invasion zone; nf, nitrogen fixing zone). Bar, 200 μm. (f) High‐resolution image of bacteroids (b) housed in symbiosomes; the symbiosome membrane separating it from the cytoplasm (c) is marked with arrows, and the peribacteroid space by an asterisk (*). Bar, 500 nm.

By contrast, *Pentaclethra macroloba*, which is placed among the first‐branching lineages of the mimosoid clade (Fig. 1), has indeterminate nodules (Fig. 3a) with infected cells in the N_2_‐fixing zone surrounded by uninfected cells (Fig. 3b) containing bacteroids that are not surrounded by a cell wall (Fig. 3c) but are clearly enclosed within symbiosomes (Fig. 3d). In the same clade, *Xylia xylocarpa* also has SYM‐type nodules (Fig. S1g,h). *Chidlowia sanguinea* nodules are similar to those of *P. macroloba*, except that the IZ is more prominent (Fig. 3e); the bacteroids are also enclosed in symbiosomes (Fig. 3f). *Chidlowia sanguinea* is sister to a large clade containing the bulk of mimosoid species, wherein SYM‐type nodules are consistently present, as illustrated by *Entada polystachya* (Fig. S1a,b), *Enterolobium cyclocarpum* (Fig. S1c,d) and *Lachesiodendron viridiflorum* (Fig. S1e,f), showing that symbiosomes are universally found in nodulating lineages across the entire Mimosoid clade (Fig. 1; Table S1).

**Fig. 4:**
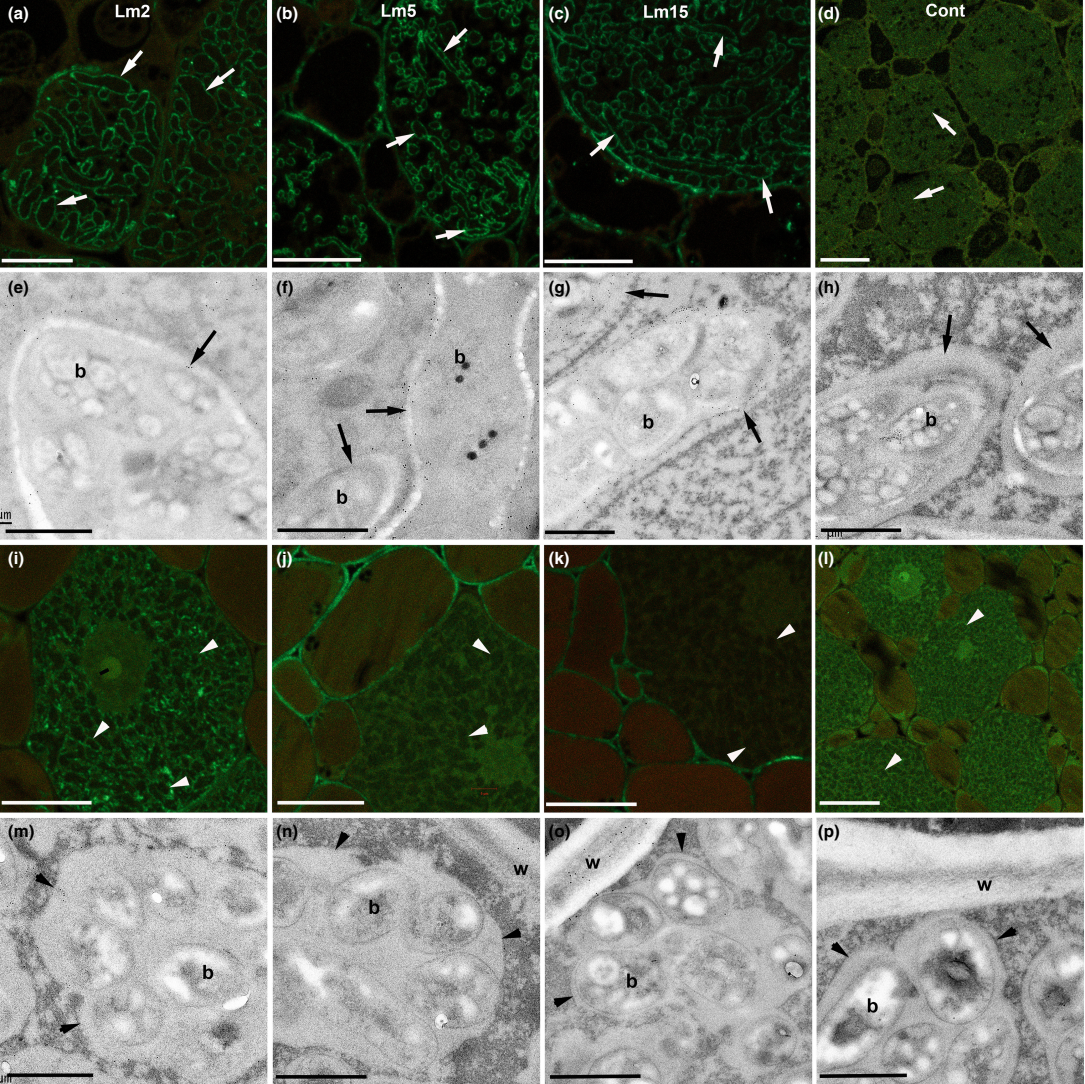
Fixation threads (FTs) contain cell wall components in addition to unesterified (JIM5) and esterified (JIM7) pectin. Confocal laser scanning microscopy (CLSM) with anti‐rat Alexa Fluor 488 (a–d, i–l) and immunogold transmission electron microscopy with anti‐rat 10 nm gold (e–h, m–p) of *Erythrophleum* (a–h) and *Pentaclethra* (i–p) nodules incubated in monoclonal antibodies raised in rat against various plant cell wall components: Lm2 (a, e, i, m), which labels arabinogalactose protein (AGP) glycan; Lm5 (b, f, j, n), which labels the pectic polysaccharide rhamnogalacturonan; and Lm15 (c, g, k, o), which labels the XXXG motif of the nonpectic, noncellullosic polysaccharide xyloglucan. Control sections incubated in buffer alone without a primary antibody are presented in (d, h, l, p). FTs are indicated by arrows in (a–h), and symbiosomes by arrowheads in (i–p). w, host cell wall separating plant cells; b, bacteroid. Bars: (a–d, i–l) 5 μm; (e–h, m–p) 1 μm.

The FT wall in *Erythrophleum* nodules was investigated further using monoclonal antibodies against AGP glycan (Lm2), the pectic polysaccharide rhamnogalacturonan (Lm5) and the nonpectic, noncellullosic polysaccharide xyloglucan (Lm15). These probes were capable of clearly delineating FTs by both CLSM (Fig. 4a–d) and TEM (Fig. 4e–h), indicating that the walls of FTs contain all three of these components. By contrast, the symbiosomes in *Pentaclethra* nodule sections treated identically were very difficult to discern using CLSM (Fig. 4j–l), and although they could be observed under TEM they had few or no gold particles associated with them, indicating an absence of cell wall components (Fig. 4n–p). The exception was Lm2 (AGP glycan), which labelled *Pentaclethra* symbiosomes (Fig. 4i,m), and thus confirmed previous observations of AGP in the symbiosome membrane made with pea (*Pisum sativum* L.) nodules (Tsyganova *et al*., 2021).

### Phylogeny and evolution of nodule types

Ancestral estimation of nodulation and nodule types across the caesalpinioid phylogeny reveals two independent transitions from FT‐ to SYM‐type nodules, first on the branch subtending the Mimosoid clade in the mid‐Eocene, 46–41 million years ago (Ma) (95% highest posterior density (HPD): 46.5–40.7 Ma), and later within the genus *Chamaecrista* (P. A. Casaes *et al*., unpublished) in the early to mid‐Miocene, 27–13 Ma (95% HPD: 27.6–12.4 Ma) (Fig. 1), or potentially later given our sparse sampling of species of *Chamaecrista*. Seventeen losses of nodulation are hypothesized across Caesalpinioideae, 12 in ancestrally FT‐type lineages and five in ancestrally SYM‐type lineages (Fig. 1; Table S3), implying rates of losses of 0.0174 per million years (ancestrally FT‐type) and 0.0028 (ancestrally SYM‐type), that is up to six times greater for FT‐type lineages than SYM‐type lineages (Fig. 1; Table S4), suggesting that nodulation based on FT‐type nodules is significantly more prone to loss than nodulation based on SYM‐type nodules. Additional analyses show that allowing all transitions between character states results in an identical reconstruction of losses (Fig. S3). Alternative scoring of character states for taxa with missing data (e.g. assigning equal weight to nodulation and nonnodulation states), or inclusion of losses within *Senegalia* do not significantly affect the outcome (Figs S4, S5). All this suggests that the data strongly support our model of evolution of nodule types across Caesalpinioideae and the higher rate of losses associated with FT‐type lineages (Fig. 1).

Maximum ages of evolutionary losses of nodulation from FT‐type nodule ancestry span the late Palaeocene to the late Oligocene (59–24 Ma (95% HPD: 59.2–23.0 Ma)) and from SYM‐type nodule ancestry from the late Eocene to the late Oligocene (37–25 Ma (95% HPD: 37.2–24.6 Ma)) (Fig. 1; Table S3).

## Discussion

Within legumes, nodulation is restricted to the two largest subfamilies, Caesalpinioideae (*sensu* LPWG, 2017) and Papilionoideae. The idea that nodulation was ‘stabilized’ in these two lineages was noted by Werner *et al*. (2014), who referred to core clades of Papilionoideae as ‘stable fixers’ (i.e., a clade across which losses of nodulation were almost absent) and to the Mimosoid clade (subfamily Mimosoideae in Werner *et al*., 2014) as having a ‘moderately stable fixing state’, where losses of nodulation were infrequent. Here we provide an explanation for this pattern: that the evolution of SYM‐type nodules, with bacteroids contained within a symbiosome, accounts for this greater stability. FT‐type nodules characterize almost all nodulating nonmimosoid Caesalpinioideae genera, while the transition to SYM‐type nodules on the stem lineage of the Mimosoid clade (Fig. 1) coincides with a shift to fewer losses of nodulation, and to a significantly lower rate of losses of nodulation per million years (i.e. a shift to greater evolutionary stability of N_2_‐fixation in this clade). As the FT‐type nonmimosoid Caesalpinioideae lineages are relatively more densely sampled in our phylogeny (Fig. 1) than the SYM‐type mimosoids, the difference between the rates of loss of FT and SYM is probably underestimated; the difference in rates of losses from FT‐type lineages compared to SYM‐type lineages is likely to be even greater than we report here.

In Papilionoideae, the sister group of Caesalpinioideae (LPWG, 2017; Koenen *et al*., 2020b), nodulating and nonnodulating genera appear to be similarly concentrated phylogenetically across the initial divergences of the subfamily, while later stabilizing as nodulating in most lineages within the large 50 kb inversion clade (Werner *et al*., 2014; Doyle, 2016; Epihov *et al*., 2017; van Velzen *et al*., 2018a). It remains to be tested whether there is a similar association between more frequent losses of nodulation and FT‐type nodules in Papilionoideae, where FT‐type nodules are also found sporadically, but the vast majority of nodulating lineages and all the species‐rich nodulating clades – the ‘stable fixers’ (Werner *et al*., 2014) – have SYM‐type nodules. Lack of phylogenetic resolution among the initial divergences in Papilionoideae (Cardoso *et al*., 2012, 2013; LPWG, 2017) means that this test must await a more robust phylogeny.

Assuming a single gain of nodulation followed by massive losses, the consistent occurrence of FT‐type nodules across nodulating lineages in the nonmimosoid grade of Caesalpinioideae demonstrates that FT‐type nodules are ancestral within Caesalpinioideae and persisted through early evolution of that subfamily (Fig. 1). The FT‐type nodule also characterizes actinorhizal nodules (Pawlowski & Demchenko, 2012) and rhizobial nodules in *Parasponia* (Lancelle & Torrey, 1985) and is thus most likely to be ancestral across the N_2_‐fixing clade (Shen & Bisseling, 2020) and legumes as a whole. As noted by Koenen *et al*. (2020b), rapid initial divergence of the six legume subfamilies implies additional losses of nodulation along the stem lineages or early in the crown group divergences of subfamilies Cercidoideae, Detarioideae, Dialioideae and Duparquetioideae, or potentially even more numerous parallel losses in more recent times, as documented within the genus *Trema* (Cannabaceae) (van Velzen *et al*., 2018a), as no extant members of these subfamilies are known to nodulate. Although we have not looked at actinorhizal symbioses here, the very low proportion of nodulated species within Cucurbitales, Fagales and Rosales (Ardley & Sprent, 2021) suggests that many lineages have suffered losses of nodulation similar to the Cercidoideae, Detarioideae, Dialioideae and Duparquetioideae, and the nonmimosoid Caesalpinioideae. However, although they might also be considered as housing their symbionts within FTs, the filamentous nature of *Frankia* precludes it from forming symbiosomes. Furthermore, the ability of *Frankia* to fix N_2_ *ex planta* and regulate gaseous and nutrient exchange via vesicles in nodules (*Casuarina* and *Allocasuarina* in the Fagales being the exceptions in that *Frankia* does not form vesicles in the nodules, and the host regulates gaseous exchange) means that *Frankia* has a fundamentally different relationship with its host compared to that between rhizobia and legumes (Pawlowski & Demchenko, 2012; Ardley & Sprent, 2021). Although losses of nodulation amongst actinorhizal lineages are the result of evolutionary drivers that are not currently well understood, it is possible that intimacy (or lack thereof) in terms of the interface between the two symbionts is not an important factor.

It is well established that N_2_‐fixation is energy‐demanding, limited by photosynthesis, and confers fitness advantages only when nitrogen is limiting and when the benefits derived from greater availability of nitrogen (e.g. in fostering higher photosynthetic rates in N_2_‐fixing plants) are greater than the costs of photosynthetic carbon (McKey, 1994; Hoffman *et al*., 2014; Taylor & Menge, 2018; van Velzen *et al*., 2018a). Additionally, there is experimental evidence showing that legumes increase N_2_‐fixation at elevated CO_2_ levels and that nitrogenase activity declines rapidly above 35°C and below 25°C (Trinick, 1980). Taken together, this suggests a greater advantage in being an N_2_‐fixer under early Cenozoic CO_2_ levels and temperatures (Rogers *et al*., 2009; Chen & Markham, 2021), and for those advantages to be preferentially retained in the tropics and subtropics, where FT‐type nodulators are largely restricted. Falling atmospheric CO_2_ levels and temperatures through the Cenozoic could have triggered global evolutionary losses of nodulation across the N_2_‐fixing clade (as suggested by van Velzen *et al*., 2018a) and we show that maximum ages of losses of nodulation across Caesalpinioideae are widely scattered from the late Palaeocene to the late Oligocene, 59–24 Ma (Fig. 1). However, it is important to consider that these are maximum ages rather than precise indicators of the timing of losses, and furthermore, the number of estimated losses per lineage is the minimum number of losses to explain the observed pattern at the tips, given the taxa sampled here.

It has long been recognized that evolutionary conflicts arise between hosts and symbionts over symbiont mixing, proliferation and transmission (Frank, 1996) because the presence of multiple, genetically heterogeneous symbiont strains within a host will cause symbionts to evolve traits that increase symbiont proliferation, competition and conflict, but decrease the efficiency of the symbiosis (Frank, 1996). There is clear evidence that this happens in the rhizobia–legume symbiosis (Oono *et al*., 2009; Sachs *et al*., 2018). In response to such conflicts, hosts have evolved ways to control symbiont proliferation (i.e. terminally differentiated bacteroids lose their ability to reproduce) (Mergaert *et al*., 2006; De La Peña *et al*., 2018; Ardley & Sprent, 2021), discriminate among symbionts (Yang *et al*., 2017; De La Peña *et al*., 2018; Ardley & Sprent, 2021) and penalize noncooperating symbionts (Kiers *et al*., 2003; Oono *et al*., 2009; Ardley & Sprent, 2021). All these approaches are more effective when symbionts are compartmentalized within hosts, and all occur in the rhizobia–legume symbiosis (Sachs *et al*., 2018), although almost all evidence is from SYM‐type papilionoids. We suggest that the anatomical differences between legume FTs and SYMs represent different degrees to which symbionts are effectively compartmentalized, as argued by Chomicki *et al*. (2020). The SYM‐type nodule, in which the microsymbiont is released from the wall‐bound IT into membrane‐bound symbiosomes within the host cell, allows for a more intimate and potentially more effective and enduring symbiotic partnership where the plant has invested in the establishment of an N_2_‐fixing ‘organelle’, which Parniske (2018) argued has only occurred in legumes and in the *Gunnera*–*Nostoc* symbiosis. In SYM‐type nodules, the plant host assumes greater control of the microsymbiont and supplies more of the components required for bacteroid metabolism and N_2_‐fixation (Hakoyama *et al*., 2009; Udvardi & Poole, 2013). This more intimate endosymbiosis reaches its pinnacle in the Inverted Repeat Lacking Clade (IRLC) of Papilionoideae, in which swollen endoreduplicated bacteroids that have lost their capacity for free‐living growth, but which are highly efficient at fixing N, are prevalent (Oono *et al*., 2009; Ardley & Sprent, 2021). The bacteroid in the FT, although surrounded by a membrane analogous to the SYM membrane, remains surrounded by a cell wall, albeit a thin one which contains little pectin, which means that it is extracellular (i.e. in the apoplast). Thus, while FTs represent a modest degree of compartmentalization, at least in *Parasponia* there is evidence to suggest that FTs are not effective in controlling growth of inefficient rhizobial strains (Op den Camp *et al*., 2012). These differing degrees of compartmentalization provide a compelling reason why SYM‐type nodulators, especially those in the IRLC (Westhoek *et al*., 2021), are less likely to be affected by cheating or infiltration by inefficient microsymbionts compared to FT‐type nodulator hosts, which remain in a ‘looser’ relationship with their symbionts.

It might be assumed that the wall of the FT would also present an additional barrier in terms of nodule O_2_ relationships and host–symbiont nutrient exchange. However, the FT wall is thin, consisting mainly of cellulose, hemicelluloses and AGP, but with reduced levels of homogalacturonan (HG) pectin components compared to the thicker and stiffer IT wall (this study), and hence presumably relatively permeable to C4‐dicarboxylates and ammonia (Brewin, 2004). The presence of abundant leghaemoglobin (Lb) suggests that rhizobial FT nodules are not obviously different from SYM‐type nodules in their O_2_ exchange. We propose that the role of the thin‐walled FT appears to be protective; that is, it prevents the undifferentiated bacteroids going ‘rogue’ and proliferating within the host cell, but in parallel, prevents the plant from identifying them as pathogens and attacking them. In all other respects, FT‐type nodules are similar to SYM‐types in still possessing a symbiosome membrane which surrounds the FT and is the real interface of nutrient exchange between the two partners. In short, the FT represents an example of a symbiosis that functions well enough to benefit both partners, but is a looser, more metabolically independent association.

SYM‐type nodules may accommodate a larger diversity of symbionts and facilitate adaptation to temperate climates. This is in line with the greater diversity of α‐ and β‐rhizobial types in SYM‐type nodules and the wider geographical distribution and environmental span of SYM‐type nodulating legumes (Sprent *et al*., 2017; Ardley & Sprent, 2021), compared to FT‐type nodules whose microsymbionts appear to be largely limited to *Bradyrhizobium* (Fonseca *et al*., 2012; Parker, 2015; Ardley & Sprent, 2021) and which are mostly confined to the tropics and subtropics where bradyrhizobia are dominant and widespread (Parker, 2015; Meng *et al*., 2019).

Despite the significantly lower rates of loss of nodulation in mimosoids with SYM‐type nodules compared to FTs in the remaining Caesalpinioideae, SYM‐type nodulation is not impervious to evolutionary loss. Our reconstruction documents five losses of nodulation within the Mimosoid clade: on the branches subtending the *Amblyogonocarpus* + *Adenanthera* clade, and the genera *Parkia*, *Cylicodiscus*, *Newtonia* and *Zapoteca* (Fig. 1; Table S3). These losses in ancestrally SYM‐type lineages suggest that even the advantages conferred by stricter SYM‐type compartmentalization can be insufficient to prevent losses of nodulation. Within the genus *Senegalia*, a small subset of species have apparently lost the ability to nodulate (Sprent, 2001, 2009). Current phylogenies imply that nodulation has been lost in species of each of the two main subclades within *Senegalia*, offering a useful system for understanding environmental or other factors associated with loss of nodulation.

### Conclusions

The evolution of the symbiosome in species‐rich nodulating legume lineages offers a compelling explanation for the well‐known but poorly understood highly uneven distribution of nodulating species richness across the N_2_‐fixing clade. While nodulation has been suggested as a possible key innovation underpinning the evolutionary success of legumes, our results suggest that it was adoption of SYM‐type nodules and the innovation of the symbiosome that underpinned the stabilization of N_2_‐fixation and potentially contributed to massive diversification of species within Caesalpinioideae and Papilionoideae, the two most diverse and geographically widespread subfamilies of legumes. Furthermore, the greater propensity of the FT‐type nodule to be secondarily lost and for SYM‐type lineages to persist and diversify provides a potent example of the long‐term evolutionary benefits and outcomes of stricter compartmentalization in symbiotic cooperation, as suggested by Chomicki *et al*. (2020).

We show that the grade of Caesalpinioideae lineages subtending the Mimosoid clade is a hotspot of evolutionary transitions between phylogenetically intermingled nodulating and nonnodulating lineages (Fig. 1), including two independent transitions from FT‐ to SYM‐type nodules as well as numerous losses of nodulation. The phylogeny and detailed evolutionary trajectories of nodulation and nodule anatomies presented here provide a robust framework for comparative genomic analyses of FT and SYM nodulating and nonnodulating lineages across Caesalpinioideae. This includes *Chamaecrista*, the most species‐rich genus in the nonmimosoid grade of Caesalpinioideae, and the only nodulating genus in the subfamily which contains species with both SYM‐and FT‐type nodules (Naisbitt *et al*., 1992), thus representing a second possible transition from FT to SYM.

## Author contributions

EJMK, JJR, CEH and EKJ designed the study and interpreted the results, JJR carried out the phylogenetic analysis, SMF, EKJ, EG, KMW and YP performed anatomical analyses, SMF, EKJ, EG, DC, GKDA, J Akomatey, NT, HSG, YP, MM, NT, PS, HCL and CEZ sampled nodules in the field, and SMF, EJMK, JJR, EKJ, J Ardley, DC, EG, YP, JIS and CEH wrote the paper.

## Supporting information


**Fig. S1** Symbiosomes are standard in nodules of caesalpinioids from the Mimosoid clade.
**Fig. S2** Fixation threads (FTs) are standard in nonmimosoid grade Caesalpinioid nodules.
**Fig. S3** Evolutionary trajectory of nodulation and nodule type when transitions between all nodulation states are allowed. Methods and legend otherwise as for Fig. 1.
**Fig. S4** Evolutionary trajectory of nodulation and nodule type when taxa with missing data have been assigned equal weight to nodulation and nonnodulation states.
**Fig. S5** Evolutionary trajectory of nodulation and nodule type when two additional losses of nodulation within *Senegalia* are included.
**Notes S1** References for Table S2.

**Table S1** Caesalpinioideae and outgroup taxa used in the time‐calibrated phylogeny depicting evolutionary trajectories of nodulation and nodule type.
**Table S2** Occurrence of fixation threads (FTs) and/or symbiosomes in nodules from Caesalpinioideae (Mim, belongs to the Mimosoid clade) extracted from the literature and from the unpublished observations of the authors.
**Table S3** Type, location and age of transitions in nodulation status as depicted in Fig. 1.
**Table S4** Summary of rates of losses of nodulation per million years across analyses.
Please note: Wiley Blackwell are not responsible for the content or functionality of any Supporting Information supplied by the authors. Any queries (other than missing material) should be directed to the *New Phytologist* Central Office.

## Data Availability

The data that support the findings of this study are available in the Supporting Information of this article.
