# Supplementary material for: The innovation of the symbiosome has enhanced the evolutionary stability of nitrogen fixation in legumes
Source: New Phytol. 2022 Jul 28;235(6):2365–77. doi: 10.1111/nph.18321 (PMC9541511; doi:10.1111/nph.18321)
Supplement: Supplementary file 1 — Fig. S1 Symbiosomes are standard in nodules of caesalpinioids from the Mimosoid clade. Fig. S2 Fixation threads (FTs) are standard in nonmimosoid grade Caesalpinioid nodules. Fig. S3 Evolutionary trajectory of nodulation and nodule type when transitions between all nodulation states are allowed. Methods and legend otherwise as for Fig. 1. Fig. S4 Evolutionary trajectory of nodulation and nodule type when taxa with missing data have been assigned equal weight to nodulation and nonnodulation states. Fig. S5 Evolutionary trajectory of nodulation and nodule type when two additional losses of nodulation within Senegalia are included. Notes S1 References for Table S2. [file NPH-235-2365-s001.pdf]

New Phytologist Supporting Information

The innovation of the symbiosome has enhanced the evolutionary stability of nitrogen fixation in legumes

Sergio M. de Faria, Jens J. Ringelberg, Eduardo Gross, Erik J.M. Koenen, Domingos Cardoso, George K.D. Ametsitsi, John Akomatey, Marta Maluk, Nisha Tak, Hukam S. Gehlot, Kathryn M. Wright, Neung Teaumroong, Pongpan Songwattana, Haroldo C. de Lima, Yves Prin, Charles E. Zartman, Janet I. Sprent, Julie Ardley, Colin E. Hughes, and Euan K. James

Accepted 31 May 2022

Notes S1. References for Table S2.

Baird LM, Virginia RA, Webster BD (1985) Development of Root Nodules in a Woody Legume, *Prosopis glandulosa* Torr. Botanical Gazette 146: 39-43.

Beukes CW, Boshoff FS, Phalane FL, Hassen AI, le Roux MM, Stepkowski T, Venter SN, Steenkamp ET (2019) Both alpha and beta-rhizobia occupy the root nodules of *Vachellia karroo* in South Africa. Front Microbiol 10:1195.

Bontemps, C., Rogel, M.A., Wiechmann, A., Mussabekova, A., Moody, S., Simon, M.F., Moulin, L., Elliott, G.N., Lacercat-Didier, L., Dasilva, C., Grether, R., Camargo-Ricalde, S.L., Chen, W., Sprent, J.I., Martínez-Romero, E., Young, J.P.W., and James, E.K. (2016) Endemic *Mimosa* species from Mexico prefer alphaproteobacterial rhizobial symbionts. *New Phytologist* **209,** 319–333.

Bournaud C., de Faria, S.M., dos Santos, J.M.F., Tisseyre, P., Silva, M., Chaintreuil, C., Gross, E., James, E.K., Prin, Y., and Moulin, L. (2013) *Burkholderia* species are the most common and preferred nodulating symbionts of the Piptadenia Group (tribe Mimoseae). *PLoS ONE* **8,** 63476.

Bournaud C., James, E.K., de Faria, S.M., Lebrun, M., Melkonian, R., Duponnois, R., Tisseyre, P., Moulin, L. and Prin, Y. (2018) Interdependency of efficient nodulation and arbuscular mycorrhization in *Piptadenia gonoacantha,* a Brazilian legume tree. *Plant, Cell and Environment* **41,** 2008 – 2020.

Canosa GA, de Faria SM, de Moraes LFD (2012) Leguminosas florestais da Mata Atlántica brasileira fixadoras de nitrogênio atmosférico. Embrapa Communicado Técnico 144 ISSN 1517-8862

Chen, W-M., James, E.K., Prescott, A.R., Kierans, M., and Sprent, J.I. (2003) Nodulation of *Mimosa* spp. by the β-proteobacterium *Ralstonia taiwanensis. Molecular Plant-Microbe Interactions* **16**, 1051-1061.

Chen, W-M., de Faria, S.M., Straliotto, R., Pitard, R.M., Simões-Araùjo, J.L., Chou, Yi-Ju Chou, J-H., Barrios, E., Prescott, A.R., Elliott, G.N., Sprent, J.I., Young, J.P.W., and James, E.K. (2005) Proof that *Burkholderia* Forms Effective Symbioses with Legumes: a Study of Novel *Mimosa*-nodulating Strains from South America. *Applied and Environmental Microbiology* **71,** 7461-7471.

Chen, W-M., James, E.K., Chou, J-H, Sheu, S-Y., Yang, S-Z., and Sprent, J.I. (2005) Beta-rhizobia from *Mimosa pigra*, a newly-discovered invasive plant in Taiwan. *New Phytologist* **168**, 661-675.

Choudhary, C., Tak, N., Bissa, G., Chouhan, B., Choudhary, P., Sprent, J.I., James, E.K., and Gehlot H.S. (2020) The widely distributed legume tree *Vachellia* (*Acacia*) *nilotica* subsp. *indica* is nodulated by genetically diverse *Ensifer* strains in India. *Symbiosis* **80,** 15-31.

Cordero I, Ruiz-Díez B, Coba de la Peña T, Balaguer L, Lucas MM, Rincón A, Pueyo JJ (2016) Rhizobial diversity, symbiotic effectiveness and structure of nodules of *Vachellia macracantha*. Soil Biol Biochem 96: 39-54. doi.org/10.1016/j.soilbio.2016.01.011

Diabate M, Munive A, Faria SM de, Ba A, Dreyfus B, Galiana A (2005) Occurrence of nodulation in unexplored leguminous trees native to the West African tropical rainforest and inoculation response of native species useful in reforestation. New Phytol 166: 231–239.

Dupuy, N (1993) Contribution à l'étude de la symbiose fixatrie d'azote entre *Acacia albida* et *Bradyrhizobium* sp. Thèse de doctorat. Université de Lille.

Elliott, G.N., Chen, W-M., Chou, J-H., Wang, H-C., Sheu, S-Y., Perin, L., Reis, V.M., Moulin, L., Simon, M.F., Bontemps, C., Sutherland, J.M., Bessi, R., de Faria, S.M., Trinick, M.J.,

Prescott, A.R., Sprent, J.I. and James, E.K. (2007) *Burkholderia phymatum* is a highly effective nitrogen-fixing symbiont of *Mimosa* spp. and fixes nitrogen *ex planta*. *New Phytologist* **173,** 168-180.

Elliott, G.N., Chou, J-H., Chen, W-M., Bloemberg, G.V., Bontemps, C., Martínez- Romero, E., Velázquez, E., Young, J.P.W., Sprent, J.I. and James, E.K. (2009) *Burkholderia* spp. are the most competitive symbionts of *Mimosa*, particularly under N-limited conditions. *Environmental Microbiology* **11,** 762-778.

Faria SM de, Lima HC de (1998) Additional studies of the nodulation status of legume species in Brazil. Plant and Soil 200, 185–192.

Faria SM de, Lima HC de (2002) Levantamento de nodulação em leguminosas arbóreas e arbustivas em áreas de influência da Mineração Rio do Norte – Porto Trombetas / PA. Embrapa Agrobiologia. Documentos 159. 32 pp. ISSN 1517-8498 http://www.infoteca.cnptia.embrapa.br/infoteca/handle/doc/624782

Faria SM de, Lima HC de, Ribeiro RD, Castilho AF, Henriques JC (2006) Nodulação em espécies leguminosas da região de Porto Trombetas, Oriximiná, Estado do Pará e seu potencial uso no reflorestamento de bacias de rejeito do lavado de bauxita. Embrapa Agrobiologia. Documentos 209. 14 pp. ISSN 1517-8498 http://www.infoteca.cnptia.embrapa.br/infoteca/handle/doc/628849

Faria SM de, Diedhiou AG, Lima HC de, Ribeiro RD, G, Castilho AF, Galiana A, Henriques JC (2010) Evaluating the nodulation status of leguminous species from the Amazonian forest of Brazil. J Exp Bot 61: 3119–3127.

Faria SM de, Moraes, LFD de,Lima HC de, Ribeiro RD, Mattos, CMJ, Rodrigues TM, Castilho AF, Canosa GA, Silva MAP (2011) Composição florística de leguminosas com potencial para fixação biológica de nitrogênio em áreas de vegetação de Canga (savana metalófita) do entorno do complexo minerador de Carajás. Embrapa Agrobiologia. Communicado Técnico 140. 20 pp. ISSN 1517-8862 http://www.infoteca.cnptia.embrapa.br/infoteca/handle/doc/921085

Faria SM de, McInroy SG, Sprent JI (1987) The occurrence of infected cells, with persistent infection threads, in legume root nodules. Can J Bot 65: 553-558.

Fonseca, M.B., Peix, A., de Faria, S.M., Mateos, P.F., Rivera, L.P., Simões-Araujo, J.L., Costa França, M.G., dos Santos Isaias, R.M., Cruz, C., Velázquez, E., Scotti, M.R., Sprent, J.I. and James, E.K. (2012) Nodulation in *Dimorphandra wilsonii* Rizz. (Caesalpinioideae), a threatened species native to the Brazilian Cerrado. *PLoS ONE* **7,** e49520

Gehlot, H.S., Tak, N., Kaushik, M., Mitra, S., Chen, W-M., Poweleit, N., Panwar, D., Poonar, N., Parihar, R., Tak, A., Sankhla, I.S., Ojha, A., Rao, S.R., Simon, M.F., dos Reis Junior, F.B., Perigolo, N., Tripathi, A., Sprent, J.I., Young, J.P.W., James, E.K., and Gyaneshwar P. (2013) An invasive *Mimosa* in India does not adopt the symbionts of its native relatives. *Annals of Botany* **112:** 179-196.

Gross E, Cordeiro L, Caetano FH (2002) Nodule ultrastructure and initial growth of *Anadenanthera peregrina* (L.) Speg. var. *falcata* (Benth.) Altschul plants infected with rhizobia. Ann Bot 90:175-183. doi:10.1093/aob/mcf184

Gyaneshwar, P., Hirsch, A.M., Moulin, L., Chen, W-M., Elliott, G.N., Bontemps, C., Estrada-de los Santos, P., Gross, E., dos Reis Junior, F.B., Sprent, J.I., Young, J.P.W. and James, E.K. (2011) Legume-nodulating betaproteobacteria: diversity, host range and future prospects. *Molecular Plant-Microbe Interactions* **24,** 1276–1288.

James, E.K., Sprent, J.I., Sutherland, J.M., McInroy, S.G. and Minchin, F.R. (1992) The structure of nitrogen fixing nodules on the aquatic mimosoid legume *Neptunia plena*. *Annals of*

Botany **69**, 173-180.

Laste KCD, Gonçalves FS, Faria SM de *(*2008) Estirpes de rizóbio eficientes na fixação biológica de nitrogênio para leguminosas com potencial de uso na recuperação de áreas mineradas. Embrapa Agrobiologia. Comunicado **Técnico, 115**, 8 pp. ISSN 1517-8862 http://www.infoteca.cnptia.embrapa.br/infoteca/handle/doc/629821

Moulin L., Klonowska, A., Bournaud, C., Booth, K., Vriezen, J.A.C., Melkonian, R., James, E.K., Young, J.P.W., Bena, G., Hauser, L., Land, M., Kyrpides, N., Bruce, D., Chain, P., Copeland, A., Pitluck, S., Woyke, T., Lizotte-Waniewski, M., Bristow, J., Riley, M. (2014) Complete Genome sequence of *Burkholderia phymatum* STM815^T, a broad host range and efficient nitrogen-fixing symbiont of *Mimosa* species. *Standards in Genomic Sciences* **9,** 763-774.

Naisbitt, T., James, E.K. and Sprent, J.I. (1992) The evolutionary significance of the legume genus *Chamaecrista*, as determined by nodule structure. *New Phytologist* **122**, 487-492.

Perrineau MM, Galiana A, de Faria SM, Bena G, Duponnois R, Reddell R, Prin Y (2012) Monoxenic nodulation process of *Acacia mangium* (Mimosoideae, Phyllodineae) by *Bradyrhizobium* sp. Symbiosis 56:87-95. doi:10.1007/s13199-012-0163-5

Platero, R., James, E.K., Rios, C., Iriarte, A., Sandes, L., Zabaleta, M., Battistoni, F., and Fabiano, E. (2016) Novel *Cupriavidus* strains isolated from root nodules of native Uruguayan *Mimosa* species. *Applied and Environmental Microbiology* **82,** 3150 –3164.

Rathi, S., Tak, N., Bissa, G., Chouhan, B., Ojha, A., Adhikari, D., Barik, S.K., Satyawada, R.R., Sprent, J.I., James, E.K., and Gehlot H.S. (2018) Selection of *Bradyrhizobium* or *Ensifer* symbionts by the native Indian caesalpinioid legume *Chamaecrista pumila* depends on soil pH and other edaphic and climatic factors. *FEMS Microbiology Ecology* 10.1093/femsec/fiy180

dos Reis Junior, F.B., Simon, M.F., Gross, E., Boddey, R.M., Elliott, G.N., Neto, N.E., Loureiro, M.F., Queiroz, L.P., Scotti, M.R., Chen, W-M., Norén, A., Rubio, M.C., de Faria, S.M., Bontemps, C., Goi, S.R., Young, J.P.W., Sprent, J.I., and James, E.K. (2010) Nodulation and nitrogen fixation by *Mimosa* spp. in the Cerrado and Caatinga biomes of Brazil. *New Phytologist* **186,** 934-946.

Rhem, M.F.K., Cordeiro Silva, V., Ferreira dos Santos, J.M., Zilli, J.E., James, E.K., Simon, M.F., and Gross, E. (2021) The large mimosoid genus *Inga* Mill. (tribe Ingeae, Caesalpinioideae) is nodulated by diverse *Bradyrhizobium* strains in its main centers of diversity in Brazil. *Systematic and Applied Microbiology* **44,** 126268doi: https://doi.org/10.1016/j.syapm.2021.126268

Saad, MM, Crèvecoeur, M, Masson-Boivin, C, Perret X (2012) The Type 3 Protein Secretion System of *Cupriavidus taiwanensis* strain LMG19424 compromises symbiosis with *Leucaena leucocephala*. Appl. Environ Microbiol 78: 7476 –7479.

Sankhla, I.S., Tak, N., Meghwal, R.R., Choudhary, S., Tak, A., Rathi, S., Sprent, J.I., James, E.K. and Gehlot, H.S. (2017) Molecular characterization of nitrogen fixing microsymbionts from root nodules of *Vachellia* (*Acacia*) *jacquemontii*, a native legume from the Thar Desert of India. *Plant and Soil* **410,** 21–40

dos Santos, J.M.F., Casaes, P.A., Silva, V.C., Rhem, M.F.K., James, E.K., and Gross, E. (2017) Diverse genotypes of *Bradyrhizobium* nodulate herbaceous *Chamaecrista* (Moench) (Fabaceae, Caesalpinioideae) species in Brazil. *Systematic and Applied Microbiology* **40,** 69-79.

Silva, V.C., Casaes, P.A., Rhem, M.F.K., dos Santos, J.M.F., James, E.K., and Gross, E. (2018) Brazilian species of *Calliandra* Benth. (tribe Ingeae) are nodulated by diverse strains of *Paraburkholderia*. *Systematic and Applied Microbiology* **41,** 241-250.

Sprent JI. 2001. Nodulation in legumes. London, UK: Royal Botanic Gardens, Kew.

Subba Rao NS, Mateos PF, Baker D, Pankratz HS, Palma J, Dazzo FB, Sprent JI. 1995. The unique root-nodule symbiosis between *Rhizobium* and the aquatic legume, *Neptunia natans* (L. f.) Druce. Planta 196: 311–320.

Zilli, J.E., Pereira de Moraes Carvalho, C., Vieira de Matos Macedo, A., de Barros Soares, L.H., Gross, E., James, E.K., Simon, M.F., and de Faria, S.M. (2021) Nodulation of the neotropical legume genus *Calliandra* by Alpha or Betaproteobacterial symbionts is dependent on the biogeographical origins of the host species. *Brazilian Journal of Microbiology* https://doi.org/10.1007/s42770-021-00570-8

Figure S1 (next page). Symbiosomes are standard in nodules of Caesalpinioideae from the Mimosoid clade. Light (a, c, e, g) and transmission electron microscope (TEM) (b, d, f, h) images of sections of nodules from various mimosoid nodules. a, *Entada polystachya* nodule longitudinal profile illustrating the zonation typical of an indeterminate nodule (m = meristem, iz = invasion zone, nf = nitrogen fixing zone). Bar = 50 µm. b, TEM of a *E. polystachya* nf zone cell with its N-fixing bacteroids (b) contained in symbiosomes with distinct membranes (arrows) separating them from the host cytoplasm (c). Bar = 1 µm. c, *Enterolobium cyclocarpum* nodule longitudinal profile illustrating the zonation typical of an indeterminate nodule (m = meristem, nf = nitrogen fixing zone). Bar = 200 µm. d, TEM of an *E. cyclocarpum* nf zone cell with its bacteroids (b) enclosed in symbiosomes (*) that are separated from the host cytoplasm (c) by the symbiosome membrane (arrows). An IT is also present in the cell; its wall (w) is immunogold labelled with 10 nm gold particles linked to JIM5 (arrowheads). Bar = 1 µm. e, Lachesiodendron viridiflorum nodule longitudinal profile illustrating the zonation typical of an indeterminate nodule (m = meristem, nf = nitrogen fixing zone). Bar = 200 µm. f, TEM of an L. viridiflorum nf zone cell with its bacteroids (b) enclosed in symbiosomes (*) that are separated from the host cytoplasm (c) by the symbiosome membrane (arrows). Bar = 500 nm. g, high magnification view of the nf zone of a *Xylia xylocarpa* nodule showing large bacteroid-containing cells (b) surrounded by smaller and more numerous uninfected cells (u). Bar = 25 µm. h, TEM of an *X. xylocarpa* nf zone cell with its bacteroids (b) enclosed in symbiosomes that are separated from the host cytoplasm (c) by the symbiosome membrane (arrows). Bar = 500 nm.

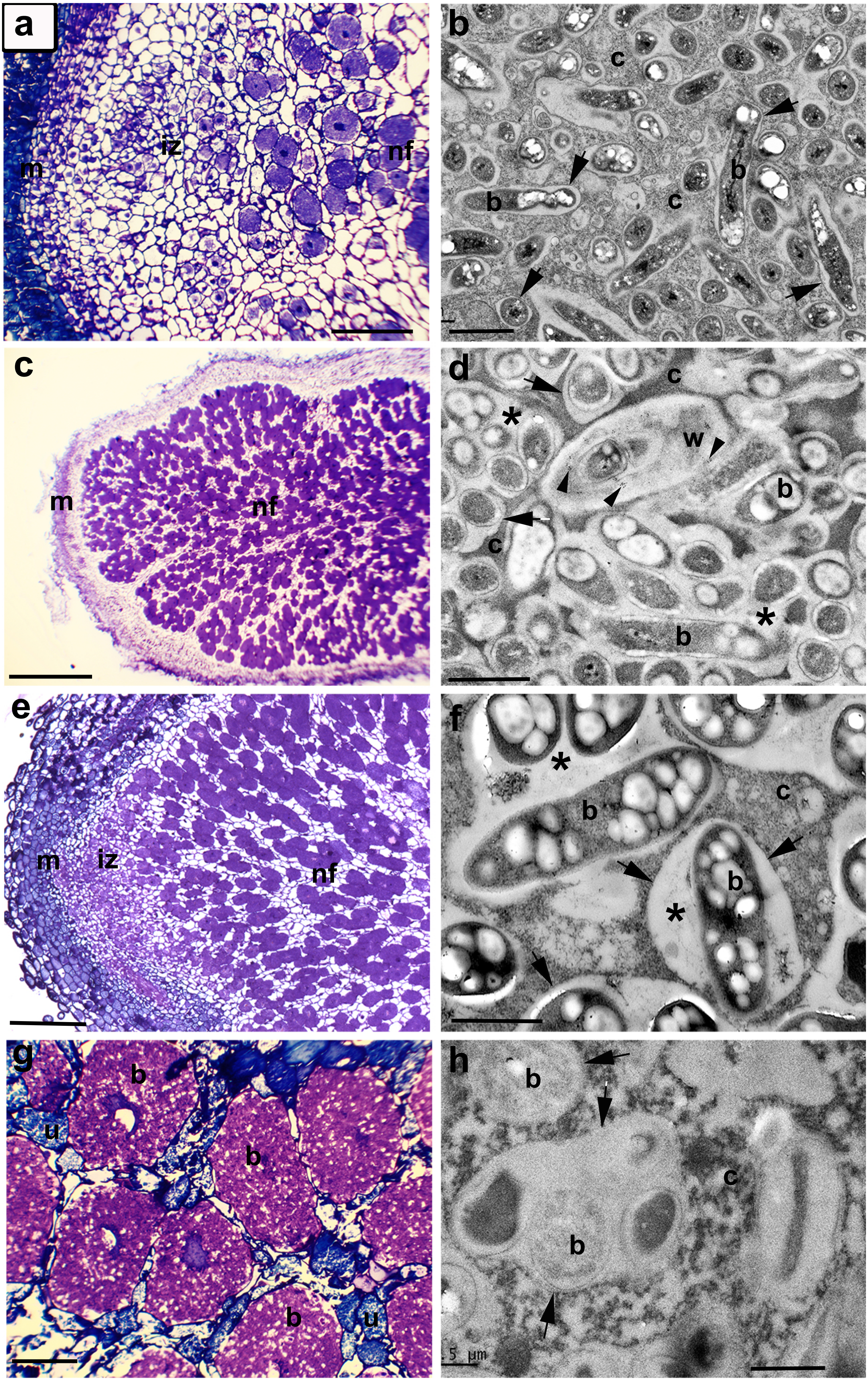
a
m
iz
nf
b
c
b
c
b
c
d
c
*
w
b
c
*
b
m
nf
e
m
iz
nf
f
*
b
c
b
*
g
b
u
b
b
u
h
b
c
b
5 µm

Figure S2 (next page). Fixation threads (FTs) are standard in non-mimosoid grade Caesalpinioideae nodules. Light (a, c, e, g, i, k) and transmission electron microscope (b, d, f, h, j, l) images of sections of nodules from *Moldenhawera* spp. (a – f), and various other caesalpinioid nodules (g – l). a, whole *M. blanchetiana* var. *multijuga* nodule longitudinal profile illustrating the zonation typical of an indeterminate nodule (m = meristem, iz = invasion zone, nf = nitrogen fixing zone). Bar = 200 μm. b, higher magnification view of the nf zone of an *M. multijuga* nodule showing large bacteroid-containing cells (b) surrounded by smaller and more numerous uninfected cells (u). Bar = 25 μm. c, *M. floribunda* iz cell with an infection thread (IT) containing a bacterium (b) within a strand of cytoplasm (c); the wall of the IT is densely immunogold labelled with 10 nm gold particles linked to JIM5 (arrows). v = vacuole. Bar = 1 μm. d, *M. blanchetiana* var. *multijuga* bacteroids (b) within FTs adjacent to the host cell wall (w) which is immunogold labelled with 10 nm gold particles linked to JIM5 (arrows); note that the FT walls are almost completely unlabelled (a few solitary gold particles are indicated by arrowheads). c = host cytoplasm. Bar = 500 nm. e, young nf cell in a *M. floribunda* nodule in which the few bacteria-containing FTs within it are still confined to strands of cytoplasm (c) adjacent to the nucleus (n). Note the strand of plasma membrane (double arrowhead) associated with an FT; it appears to be derived from the nuclear membrane (arrowheads). v = vacuole. Bar = 1 μm. f, mature FTs in the nf of a *M. floribunda* nodule. Note the thick wall (w) of the FTs and the membranes associated with them (arrows). c = cytoplasm, m = mitochondrion. Bar = 500 nm. g, high magnification view of the nf zone of a *Jacqueshuberia purpurea* nodule showing large bacteroid-containing cells (b) surrounded by smaller and more numerous uninfected cells (u). Bar = 25 μm. h, TEM of a *J. purpurea* nf zone cell with its bacteroids (b) enclosed in FTs that are separated from the host cytoplasm (c) by electron-dense walls (arrows) that are almost completely unlabelled with JIM5, except for some thickened areas (arrowheads). Bar = 500 nm. i, high magnification view of the nf zone of a *Tachigali rugosa* nodule showing large bacteroid-containing cells (b) surrounded by smaller uninfected cells (u). Bar = 25 μm. j, TEM of a *T. rugosa* nf zone cell with its bacteroids (b) enclosed in FTs that are separated from the host cytoplasm (c) by cell walls that are labelled with JIM5 (arrows). v = vacuole, m = mitochondrion. Bar = 1 μm. k, high magnification view of the nf zone of a *Campsiandra comosa* nodule showing large bacteroid-containing cells (b) surrounded by smaller uninfected cells (u). Bar = 25 μm. l, TEM of a *C. comosa* nf zone cell with its bacteroids (b) enclosed in FTs that are separated from the host cytoplasm (c) by cell walls that are labelled with JIM5 (arrows). Bar = 500 nm.

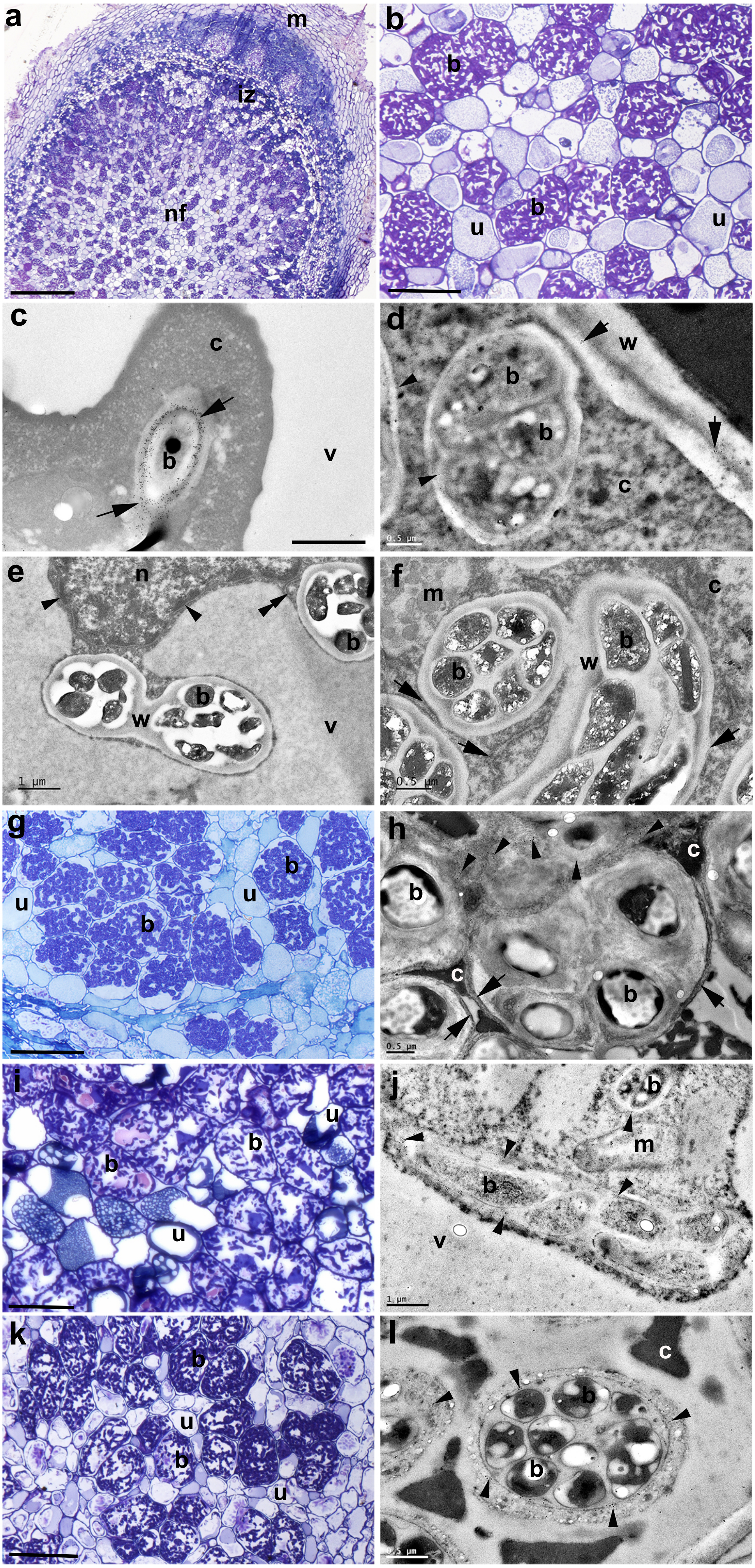

a
m
iz
nf
b
b
b
u
u
c
c
b
v
d
w
b
b
c
0.5 µm
e
n
b
b
w
v
1 µm
f
m
c
b
b
w
0.5 µm
g
b
u
u
b
h
c
b
c
b
0.5 µm
i
u
b
b
u
j
b
m
b
v
1 µm
k
b
u
b
u
l
c
b
b

Figure S3 (next page). Evolutionary trajectories of nodulation and nodule type across a time-calibrated phylogeny of the legume subfamily Caesalpinioideae using an unconstrained model that allows transitions between all nodulation states. Pie charts on nodes show the proportions of the most likely reconstructed character states: non-nodulating, fixation thread (FT-type nodules), symbiosome (SYM-type nodules), nodulating but of unknown type, and nodulation status unknown, summarised over 500 simulations. Branch colours denote the nodulation status of the node or tip it subtends and the coloured boxes in front of each taxon name show the character state for that species. The dashed orange, blue, and dark green vertical lines show the phylogenetic locations and maximum ages of the various character state transitions on the tree. Using the same colours, the histograms show the frequencies of the number of transitions from FT to SYM (blue), from SYM to non-nodulation (green) and from FT to non-nodulation (orange), and the rates of losses of nodulation per million years for SYM to non-nodulation (green) and FT to non-nodulation (orange) across 500 independent character estimations. Note that while the three other character state transitions, from non-nodulating to FT or SYM-type nodules, and from SYM to FT, were allowed under our model, no such transitions are inferred to have happened. Pli = Pliocene; Ple = Pleistocene.

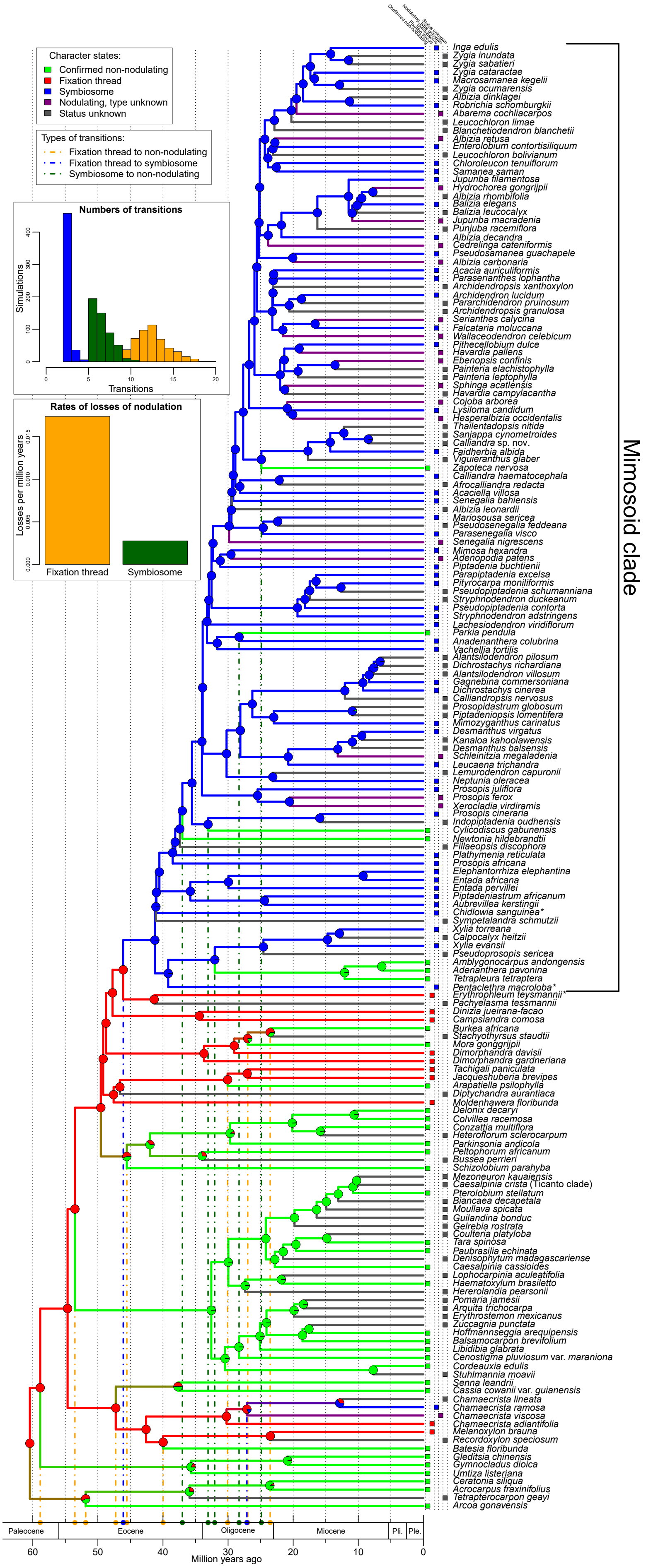

Character states:
Confirmed non-nodulating
Fixation thread
Symbiosome
Nodulating, type unknown
Status unknown
Types of transitions:
Fixation thread to non-nodulating
Fixation thread to symbiosome
Symbiosome to non-nodulating
Numbers of transitions
Simulations
Transitions
0
100
200
300
400
0
5
10
15
20
Rates of losses of nodulation
Losses per million years
0.000
0.005
0.010
0.015
Fixation thread
Symbiosome
Mimosoid clade
Inga edulis
Zygia inundata
Zygia sabatieri
Zygia cataractae
Macrosamanea kegelii
Zygia ocumarensis
Albizia dinklagei
Robrichia schomburgkii
Abarema cochliacarpos
Leucochloron limae
Blanchetiodendron blanchetii
Albizia retusa
Enterolobium contortisiliquum
Leucochloron bolivianum
Chloroleucon tenuiflorum
Samanea saman
Jupunba filamentosa
Hydrochorea gongrijpii
Albizia rhombifolia
Balizia elegans
Balizia leucocalyx
Jupunba macradenia
Punjuba racemiflora
Albizia decandra
Cedrelinga cateniformis
Pseudosamanea guachapele
Albizia carbonaria
Acacia auriculiformis
Paraserianthes lophantha
Archidendropsis xanthoxylon
Archidendron lucidum
Pararchidendron pruinosum
Archidendropsis granulosa
Serianthes calycina
Falcataria moluccana
Wallaceodendron celebicum
Pithecellobium dulce
Havardia pallens
Ebenopsis confinis
Painteria elachistophylla
Painteria leptophylla
Sphinga acatlensis
Havardia campylacantha
Cojoba arborea
Lysiloma candidum
Hesperalbizia occidentalis
Thailentadopsis nitida
Sanjappa cynometroides
Calliandra sp. nov.
Faidherbia albida
Viguieranthus glaber
Zapoteca nervosa
Calliandra haematocephala
Afrocalliandra redacta
Acaciella villosa
Senegalia bahiensis
Albizia leonardii
Mariosousa sericea
Pseudosenegalia feddeana
Parasenegalia visco
Senegalia nigrescens
Mimosa hexandra
Adenopodia patens
Piptadenia buchtienii
Parapiptadenia excelsa
Pityrocarpa moniliformis
Pseudopiptadenia schumanniana
Stryphnodendron duckeanum
Pseudopiptadenia contorta
Stryphnodendron adstringens
Lachesiodendron viridiflorum
Parkia pendula
Anadenanthera colubrina
Vachellia tortilis
Alantsilodendron pilosum
Dichrostachys richardiana
Alantsilodendron villosum
Gagnebina commersoniana
Dichrostachys cinerea
Calliandropsis nervosus
Prosopidastrum globosum
Piptadeniopsis lomentifera
Mimozyganthus carinatus
Desmanthus virgatus
Kanaloa kahoolawensis
Desmanthus balsensis
Schleinitzia megaladenia
Leucaena trichandra
Lemurodendron capuronii
Neptunia oleracea
Prosopis juliflora
Prosopis ferox
Xerocladia viridiramis
Prosopis cineraria
Indopiptadenia oudhensis
Cylicodiscus gabunensis
Newtonia hildebrandtii
Fillaeopsis discophora
Plathymenia reticulata
Prosopis africana
Elephantorrhiza elephantina
Entada africana
Entada pervillei
Piptadeniastrum africanum
Aubrevillea kerstingii
Chidlowia sanguinea*
Sympetalandra schmutzii
Xylia torreana
Calpocalyx heitzii
Xylia evansii
Pseudoprosopis sericea
Amblygonocarpus andongensis
Adenanthera pavonina
Tetrapleura tetraptera
Pentaclethra macroloba*
Erythrophleum teysmannii*
Pachyelasma tessmannii
Dinizia jueirana-facao
Campsiandra comosa
Burkea africana
Stachyothyrsus staudtii
Mora gonggrijpii
Dimorphandra davisii
Dimorphandra gardneriana
Tachigali paniculata
Jacqueshuberia brevipes
Arapatiella psilophylla
Diptychandra aurantiaca
Moldenhawera floribunda
Delonix decaryi
Colvillea racemosa
Conzattia multiflora
Heteroflorum sclerocarpum
Parkinsonia andicola
Peltophorum africanum
Bussea perrieri
Schizolobium parahyba
Mezoneuron kauaiensis
Caesalpinia crista (Ticanto clade)
Pterolobium stellatum
Biancaea decapetala
Moullava spicata
Guilandina bonduc
Gelrebia rostrata
Coulteria platyloba
Tara spinosa
Paubrasilia echinata
Denisophytum madagascariense
Caesalpinia cassioides
Lophocarpinia aculeatifolia
Haematoxylum brasiletto
Hererolandia pearsonii
Pomaria jamesii
Arquita trichocarpa
Erythrostemon mexicanus
Zuccagnia punctata
Hoffmannseggia arequipensis
Balsamocarpon brevifolium
Libidibia glabrata
Cenostigma pluviosum var. maranionа
Cordeauxia edulis
Stuhlmannia moavii
Senna leandrii
Cassia cowanii var. guianensis
Chamaecrista lineata
Chamaecrista ramosa
Chamaecrista viscosa
Chamaecrista adiantifolia
Melanoxylon brauna
Recordoxylon speciosum
Batesia floribunda
Gleditsia chinensis
Gymnocladus dioica
Umtiza listeriana
Ceratonia siliqua
Acrocarpus fraxinifolius
Tetrapterocarpon geayi
Arcoa gonavensis
Paleocene
Eocene
Oligocene
Miocene
Pli.
Ple.
60
50
40
30
20
10
0
Million years ago

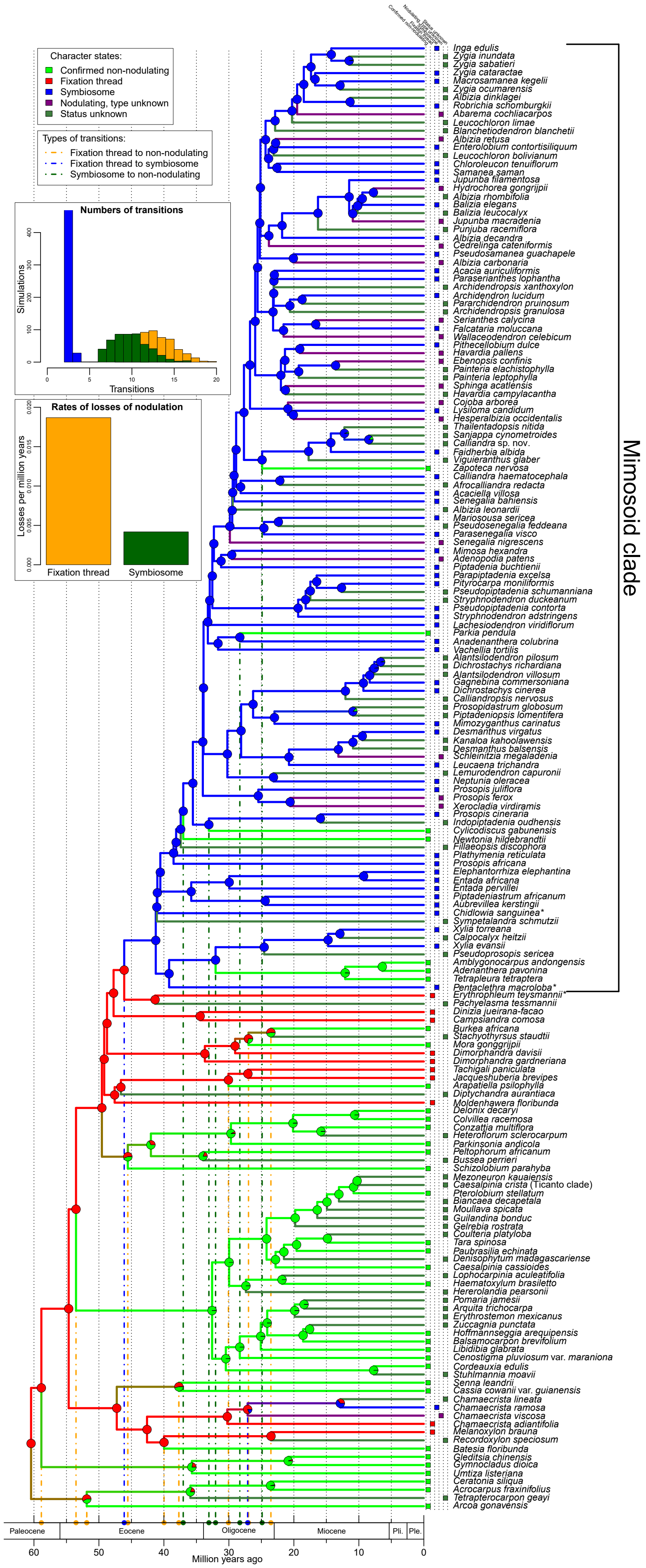

Figure S4. Evolutionary trajectory of nodulation and nodule type when taxa with missing data have been assigned equal weight to nodulation and non-nodulation states. Methods and legend otherwise as for Figure S3.

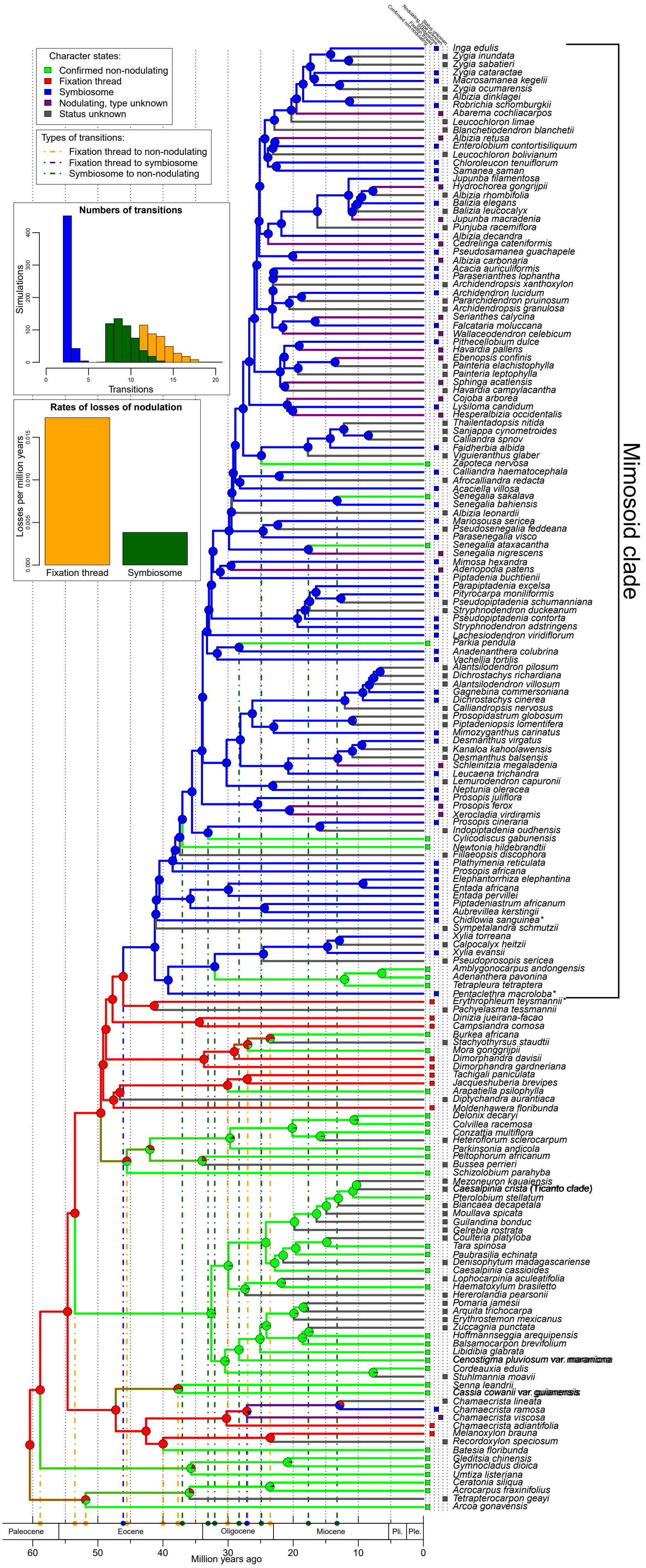

Figure S5. Evolutionary trajectory of nodulation and nodule type when two additional losses of nodulation within *Senegalia* are included. Methods and legend otherwise as for Figure S3.
